# RACK1 governs a dual metabolic switch in lung adenocarcinoma through c-Src/G6PD and TRIM21/LDHA Axes

**DOI:** 10.1038/s41419-026-08887-8

**Published:** 2026-05-29

**Authors:** Huimin Wan, Chao Li, Mengqian Xia, Lin Dong, Tingting Lin, Weiwei Guo, Fanglei Jiao, Jingjing Lu, Zhongliang Guo

**Affiliations:** 1 Department of Respiratory and Critical Care Medicine, Shanghai East Hospital, School of Medicine, Tongji University, Shanghai, China; 2 Department of General Surgery, Zhongshan Hospital, School of Medicine, Fudan University, Shanghai, China; 3 Thoracic Surgery Department, East Hospital (South), School of Medicine, Tongji University, Shanghai, China; 4 Department of Respiratory Medicine, Xiamen Humanity Hospital, Fujian Medical University, Xiamen, China; 5 Rehabilitation Medicine Center, Shanghai Pudong New District Zhoupu Hospital, Shanghai, China

**Keywords:** Cancer metabolism, Non-small-cell lung cancer

## Abstract

Lung adenocarcinoma (LUAD) remains a lethal malignancy plagued by therapy resistance and metabolic adaptability. This study identifies the scaffold protein RACK1 as a central regulator of LUAD pathogenesis and metabolic reprogramming. RACK1 is frequently upregulated in LUAD, where its expression correlates with advanced stage and poor prognosis. Mechanistically, RACK1 co-activates two pivotal metabolic pathways: it stabilizes LDHA by competing with its E3 ligase TRIM21, thereby enhancing glycolysis, and it scaffolds c-Src to phosphorylate and activate G6PD, fueling the pentose phosphate pathway. This dual metabolic switch promotes biomass production, redox balance, and drives aggressive tumor phenotypes. Crucially, combined targeting of RACK1 with c-Src or LDHA inhibition yielded synergistic anti-tumor effects in vivo, significantly surpassing monotherapies. Our findings establish RACK1 as a master metabolic regulator and propose a promising combinatorial therapeutic strategy for LUAD.

## Introduction

Lung cancer remains the leading cause of cancer-related deaths globally, with non-small cell lung cancer (NSCLC) accounting for ~85% of cases [1]. As the most prevalent NSCLC subtype, lung adenocarcinoma (LUAD) constitutes about 40% of all lung cancers and is driven by diverse genetic and epigenetic alterations affecting critical pathways, including signal transduction, DNA repair, chromatin modification, metabolism, and immune checkpoint regulation [2–5]. Although advances in low-dose computed tomography (CT) and genetic diagnostics have improved early detection, LUAD continues to contribute significantly to cancer mortality, largely due to frequent diagnosis at advanced stages and limited therapeutic efficacy [6]. Elucidating the molecular mechanisms underlying LUAD pathogenesis and metastasis is therefore essential for developing novel biomarkers and therapeutic strategies.

Metabolic reprogramming is a well-established hallmark of cancer that supports rapid tumor growth and survival through adaptive changes in glucose, lipid, and amino acid metabolism [7–10]. These shifts are regulated through signaling pathways and epigenetic mechanisms. A prominent example is the “Warburg effect”, wherein cancer cells preferentially utilize glycolysis over oxidative phosphorylation (OXPHOS) even under oxygen-sufficient conditions [11]. This metabolic switch is driven by altered expression of key enzymes—for instance, lactate dehydrogenase (LDH), which catalyzes the conversion of pyruvate to lactate [12]. In particular, lactate dehydrogenase A (LDHA), forming a homo-tetramer of subunit A, has been implicated in cancer progression and metastasis. Elevated LDHA expression correlates with poor prognosis in lung cancer [13], yet the regulatory mechanisms controlling LDHA in LUAD remain incompletely understood.

Parallel to glycolysis, the pentose phosphate pathway (PPP) serves as another crucial metabolic route in cancer cells, contributing to nucleic acid synthesis and redox homeostasis through the generation of pentose phosphates and NADPH [14]. Glucose-6-phosphate dehydrogenase (G6PD), the rate-limiting enzyme of the oxidative PPP branch, catalyzes the first step in this pathway and is frequently upregulated in LUAD, promoting proliferation, invasion, and therapy resistance [15–18]. Nevertheless, the post-translational mechanisms controlling G6PD activity in LUAD are not fully elucidated.

RACK1, a scaffolding protein belonging to the Trp-Asp (WD) repeat family, facilitates protein-protein interactions through its conserved WD40 domain [19]. It engages with diverse partners including kinases, phosphatases, receptors, G proteins, and apoptotic molecules, influencing cellular localization, activity, complex formation, and stability of its binding partners [20, 21]. Although RACK1 dysregulation has been observed in multiple cancers, its functional role and underlying mechanisms in LUAD have remained unclear.

In this study, we demonstrate that RACK1 is highly expressed in LUAD and correlates with advanced tumor stage and poor prognosis. Mechanistically, we show that RACK1 promotes glycolysis by stabilizing LDHA through competitive binding with the E3 ligase TRIM21, while simultaneously activating the PPP by scaffolding c-Src to phosphorylate and activate G6PD. This coordinated regulation of two interconnected metabolic pathways positions RACK1 as a central hub in LUAD metabolic reprogramming. Our findings identify the RACK1-LDHA/G6PD axis as a promising therapeutic target for metabolic intervention in LUAD.

## Materials and methods

### Cell culture

The human LUAD cell lines A549 (TCHu150) (RRID:CVCL_0023) and PC9 (SCSP-5085) (RRID:CVCL_B260), as well as the human embryonic kidney cell line HEK-293T (GNHu17) (RRID:CVCL_0063), were acquired from the Chinese Academy of Sciences Cell Bank. Both cell lines were authenticated by short tandem repeat (STR) profiling to confirm their identity. Cells were cultured in DMEM medium (Wisent Biotechnology, #319-027-CL) supplemented with 10% fetal bovine serum (Baidi Biotech Ltd., #F801-500) and 100 U/mL penicillin-streptomycin (Gibco, #15070063). All cell lines were maintained at 37 °C under a humidified 5% CO₂ atmosphere. To ensure cell line authenticity and absence of contamination, mycoplasma testing was performed monthly using the MycoAlert detection kit (Lonza, #LT07-710), and all cultures consistently tested negative, confirming that the cell lines were free of mycoplasma contamination for the described experiments.

### Patient samples and tissue collection

Tumor specimens (21 cases) along with matched adjacent normal tissues were obtained from patients who underwent surgical resection for LUAD at East Hospital, Tongji University between January 2022 and January 2024. Immediately following resection, fresh tissue samples were snap-frozen in liquid nitrogen. Each case was independently evaluated by two experienced pathologists to confirm diagnosis and histopathological grade. In addition, a tissue microarray (TMA) was constructed using 55 surgically resected LUAD samples collected at the same institution between January 2016 and January 2022. All tissues were fixed in 4% neutral-buffered formalin within 30 minutes after resection. Relevant clinicopathological information is provided in Table S1 and 2. The study protocol was approved by the Medical Ethics Committee of East Hospital, Tongji University (approval number: 2025YS-208). Written informed consent was obtained from all participants in accordance with the principles of the Declaration of Helsinki.

### Lentiviral constructs and transfection

Lentiviral expression vectors for Flag-tagged RACK1, Myc-tagged G6PD, His-tagged LDHA and Myc-TRIM21 were constructed by cloning the respective coding sequences into the pCDH-CMV-MCS-EF1α-Puro backbone. Plasmids encoding Src were acquired from Addgene (USA) (RRID:Addgene_42202). Short hairpin RNA (shRNA) lentiviral plasmids targeting RACK1, G6PD, LDHA, and TRIM21, as well as a non-targeting control shRNA, were obtained from Obio Technology (Shanghai, China). Lentiviruses were packaged using standard protocols and subsequently used to transduce LUAD cells. Transfection was carried out with Lipofectamine 3000 (Invitrogen, USA) in accordance with the manufacturer’s guidelines. Stable cell lines were selected and maintained under puromycin (2 μg/mL; Beyotime, ST551) treatment.

### Plasmid construction

Truncated variants of LDHA (designated LDHA-Δ1 through Δ6) were generated using Gibson Assembly. The target inserts were amplified via PCR with KOD DNA polymerase (TOYOBO, KFX-101) using appropriate template plasmids. Both the PCR products and the pCMV-based vectors were digested with EcoRI and XhoI restriction enzymes (Thermo Scientific), followed by purification with a DNA Gel Extraction Kit (Tiangen, DP103). Ligation was performed using the Gibson Assembly Master Mix (NEB, #E5510S). The assembled products were transformed into DH5α competent cells (Vazyme, C502) and plated on LB agar supplemented with ampicillin. Monoclonal colonies were screened by colony PCR with Taq Master Mix (Vazyme, P501). Positive clones were confirmed by Sanger sequencing, and validated plasmids were extracted using Miniprep Kits (Tiangen, DP105).

To introduce point mutations (Y112F, Y428F, Y507F) into human G6PD cDNA cloned into the pLenti-CMV vector, site-directed mutagenesis was carried out with the QuikChange Kit (Agilent, #200523). Mutagenic primers were used to amplify the plasmid with KOD Plus Neo DNA polymerase (TOYOBO, KOD-401). The parental template was digested with DpnI (NEB, R0176S), and the resulting mutation-bearing plasmids were transformed into XL10-Gold competent cells (Agilent, #200315). Successful mutagenesis was verified by sequencing, and endotoxin-free plasmid preparations were obtained using EndoFree Plasmid Kits (Tiangen, DP117).

### RNA isolation and quantitative real-time PCR (qPCR)

Total RNA was isolated from cultured cells and clinical tissue samples using TRIzol reagent (TaKaRa Biotechnology, Japan). First-strand cDNA was synthesized with the First Strand cDNA Synthesis Kit (Roche Diagnostics, Germany). Quantitative real-time PCR (qPCR) was carried out on the StepOnePlus™ Real-Time PCR System (Thermo Fisher Scientific, USA) with FastStar Universal SYBR Green Master Mix (Roche Diagnostics, Germany). The relative expression of target mRNAs was calculated using the ΔΔCt method and normalized to β-actin as an internal control. All primer sequences used for qPCR are listed in the Supplementary Materials (Table S3).

### Cell migration and invasion assay

Cell migration and invasion were assessed using Transwell chambers (Corning, USA). For the migration assay, 5 × 10⁴ cells in 200 µL of serum-free DMEM were seeded into the upper chamber of an uncoated insert. For the invasion assay, inserts were pre-coated with Matrigel (Corning) prior to cell seeding. The lower chamber was filled with 500 µL of DMEM supplemented with 10% FBS as a chemoattractant. After a 24-hour incubation, cells remaining on the upper membrane surface were removed by swabbing. Cells that had migrated or invaded to the lower surface were fixed with 4% paraformaldehyde, stained with 0.1% crystal violet, and quantified by counting in three random microscopic fields per insert.

### Wound healing assay

A wound healing assay was performed to assess cell migration. Briefly, after cells reached 90% confluence in a monolayer, a straight wound was created in each well by scratching with a sterile 200-µL pipette tip. The dislodged cells were removed by washing with PBS, and fresh serum-free medium was added to minimize cell proliferation. The wounds were then imaged at 0, 24, and 48 hours using a microscope. The extent of wound closure was quantified by measuring the residual wound width at each time point relative to the initial width (0 h) using ImageJ software.

### 5-ethynyl-2’deoxyuridine (EdU) assay

Cell proliferation was assessed using the 5-Ethynyl-2’-deoxyuridine (EdU) assay with the Cell-Light™ EdU DNA Cell Proliferation Kit (RiboBio, China) according to the manufacturer’s protocol. Briefly, A549 and PC9 cells were seeded in 96-well plates at a density of 1 × 10³ cells per well and allowed to adhere for 24 hours. The cells were then incubated with 50 µM EdU for 2 hours under standard culture conditions (37 °C, 5% CO₂). Following incubation, cells were fixed with 4% paraformaldehyde and permeabilized with 0.5% Triton X-100. EdU incorporation was detected using the Apollo dye, and cell nuclei were counterstained with Hoechst 33342. Fluorescence images were captured using a FV3000RS confocal microscope (Olympus, Japan).

### Western blot analysis

Total protein was extracted from cells and tissues using RIPA lysis buffer (Beyotime, China) containing protease and phosphatase inhibitor cocktails (Millipore, USA). Protein lysates were resolved by SDS-PAGE and subsequently transferred to PVDF membranes (Millipore, USA). After blocking, the membranes were incubated with specific primary antibodies overnight at 4 °C, followed by incubation with HRP-conjugated secondary antibodies. Protein bands were visualized using a Super ECL Plus kit (US Everbright Inc., China) and quantified by densitometry with β-actin serving as the loading control. Each experiment was repeated in at least three independent biological replicates. The antibodies used included the following: from Abcam, anti-β-actin (1:5000, ab8227) (RRID:AB_2305186), anti-Src (1:2000, ab109381) (RRID:AB_10865528), anti-GST (1:1000–2000, ab111947) (RRID:AB_10861266), anti-His (1:1000, ab18184) (RRID:AB_444306), anti-Flag tag (1:2500, ab205606) (RRID:AB_2916341), anti-HA tag (1: 5000, ab9110) (RRID:AB_307019), and anti-Myc (1:1000, ab9106) (RRID:AB_307014). Proteintech antibodies included anti-RACK1 (1:1000, #27592-1-AP and #66940-1-Ig) (RRID:AB_2880917 and RRID:AB_2882264), anti-LDHA (1:5000, #19987-1-AP) (RRID:AB_10646429), anti-TRIM21 (1:5000, #12108-1-AP) (RRID:AB_2209469), and anti-G6PD (1:5000, # 25413-1-AP) (RRID:AB_2880066). From Cell Signaling Technology, antibodies included anti-pan phospho-serine/threonine (1:1000, #9631) (RRID:AB_330308). For detection, HRP-conjugated secondary antibodies from Beyotime (China) were used: Goat Anti-Rabbit IgG (H + L) (1:1000, #A0208) (RRID:AB_2892644) and Goat Anti-Mouse IgG (H + L) (1:1000, #A0216) (RRID:AB_2860575).

### Immunoprecipitation

Cells were lysed on ice for 30 minutes using NP-40 lysis buffer (Beyotime Biotechnology, China). For co-immunoprecipitation (Co-IP) assays, the lysis buffer was supplemented with PhosStop phosphatase inhibitor cocktail (Roche, Switzerland) and 1 mM PMSF prior to cell lysis. Lysates were centrifuged at 12,000 × *g* for 15 minutes at 4 °C, and the resulting supernatants were incubated overnight at 4 °C with rotation in the presence of a target-specific primary antibody. Protein A/G magnetic beads (Santa Cruz Biotechnology, USA) were then added and incubated with gentle rotation overnight at 4 °C. The beads were washed five times with ice-cold wash buffer (150 mM NaCl, 50 mM Tris–HCl pH 8.0, 5 mM MgCl₂, 0.08% NP-40). After the final wash, bound proteins were eluted by heating the beads in 2× SDS-PAGE sample loading buffer (Beyotime Biotechnology) at 95 °C for 9 minutes. Eluates were analyzed by immunoblotting or liquid chromatography–tandem mass spectrometry (LC–MS/MS). All Co-IP experiments were performed in triplicate.

### Ubiquitylation assay

To assess LDHA ubiquitylation, LUAD cells stably expressing His-tagged LDHA were subjected to RACK1 knockdown or overexpression. Prior to collection, cells were treated with the proteasome inhibitor MG132 (10 μM) for 8 hours to stabilize ubiquitylated proteins. Cells were then washed three times with ice-cold phosphate-buffered saline (PBS) and lysed in a denaturing buffer containing 6 M guanidine hydrochloride, 0.1 M Na₂HPO₄/NaH₂PO₄, 10 mM Tris-HCl (pH 8.0), 5 mM imidazole, and 10 mM β-mercaptoethanol. Following centrifugation at 14,000 × *g* for 20 min at 4°C, the cleared supernatants were incubated with Ni-NTA agarose beads for 4 hours at room temperature to capture His-tagged proteins. The beads were sequentially washed with four stringent buffers: buffer A (6 M guanidine-HCl, 0.1 M phosphate buffer, 10 mM Tris-HCl, pH 8.0, 10 mM β-mercaptoethanol), buffer B (8 M urea, 0.1 M phosphate buffer, 10 mM Tris-HCl, pH 8.0, 10 mM β-mercaptoethanol), buffer C (8 M urea, 0.1 M phosphate buffer, 10 mM Tris-HCl, pH 6.3, 10 mM β-mercaptoethanol, 0.2% Triton X-100), and buffer D (8 M urea, 0.1 M phosphate buffer, 10 mM Tris-HCl, pH 6.3, 10 mM β-mercaptoethanol, 0.1% Triton X-100), with 5-minute incubations for each wash. His-tagged proteins were eluted by incubating the beads with elution buffer E (200 mM imidazole, 150 mM Tris-HCl, pH 6.7, 30% glycerol, 0.72 M β-mercaptoethanol, 5% SDS) for 30 minutes at room temperature, followed by boiling in Laemmli sample buffer at 95 °C for 10 minutes. The eluates were then analyzed by western blotting using anti-His and anti-Ubiquitin antibodies.

### GST-tagged protein purification and GST pull-down assay

Recombinant GST-tagged RACK1 was expressed in Escherichia coli BL21 transformed with the pGEX-4T-1 plasmid. Protein expression was induced with 0.4 mM isopropyl β-D-1-thiogalactopyranoside (IPTG), and GST-RACK1 was affinity-purified using glutathione-Sepharose 4B beads (GE Healthcare, UK) according to the manufacturer’s instructions. For GST pull-down assays, purified GST or GST-RACK1 bound to glutathione beads was incubated with lysates from HEK293T cells overexpressing either His-tagged LDHA or Myc-tagged G6PD. After extensive washing with GST-binding buffer to eliminate non-specific binding, interacting proteins were eluted using TNGT elution buffer. Eluates were then subjected to immunoblot analysis to detect specific protein–protein interactions. All pull-down assays were performed in three independent replicates.

### Immunofluorescence (IF) microscopy

LUAD cells were plated on confocal dishes (Biosharp) at a density of 1 × 10⁴ cells per dish. After the appropriate treatments, cells were fixed with 4% paraformaldehyde, permeabilized with 0.1% Triton X-100, and blocked with 5% BSA. Subsequently, the cells were incubated with primary antibodies against RACK1, G6PD, and LDHA overnight at 4 °C, followed by incubation with fluorophore-conjugated secondary antibodies. Nuclei were stained with DAPI. Confocal images were acquired using a FV3000RS laser scanning confocal microscope (Olympus, Japan) (RRID:SCR_017015). All experiments were repeated in at least three independent biological replicates.

### Metabolic assays

The glycolytic proton efflux rate (GlycoPER) was measured using a Glycolytic Rate Assay Kit (#103344-100, Agilent, USA). Prior to the assay, the sensor cartridge was hydrated overnight in HPLC-grade water in a non-CO₂ incubator. A phenol red-free assay medium containing 10 mM glucose, 2 mM glutamine, 1 mM pyruvate, and 5 mM HEPES was prepared and equilibrated at 37 °C under CO₂-free conditions. LUAD cells were seeded in Seahorse XF96 V3 PS Cell Culture Microplates (#101085-004, Agilent, USA) at 2 × 10⁴ cells per well and cultured overnight. Before the assay, the growth medium was replaced with the pre-warmed assay medium, and cells were incubated for 1 hour at 37 °C in a non-CO₂ incubator. Real-time glycolytic flux was analyzed using an XF96 Extracellular Flux Analyzer (Agilent, USA) (RRID:SCR_026641). To specifically assess glycolytic activity, mitochondrial respiration was inhibited using rotenone/antimycin A (Rot/AA), followed by the addition of 2-deoxy-D-glucose (2-DG) to inhibit glycolysis. All measurements were performed in triplicate and normalized to cell count.

### G6PD activity assay

G6PD enzymatic activity was measured using a commercial assay kit (BC0265, Solarbio, China). Cells were cultured in complete medium for 24 hours before being harvested. The assay is based on the oxidation of glucose-6-phosphate (G6P) by G6PD in the presence of NADP⁺, yielding 6-phosphogluconolactone and NADPH. The rate of NADPH generation, which reflects G6PD activity, was determined by monitoring the increase in absorbance at 340 nm using a microplate reader. All measurements were performed in triplicate and normalized to total protein concentration.

### NADPH/NADP+ measurement

The NADP⁺/NADPH ratio was determined using the Enhanced NADP⁺/NADPH Assay Kit with WST-8 (S0180S, Beyotime, China). Cells were seeded in 60 mm dishes and cultured for 24 hours before being lysed in 400 μL of the provided extraction buffer. For each sample, 50 μL of lysate was processed according to the manufacturer’s instructions. Absorbance was measured at 450 nm using a microplate reader maintained at room temperature. All assays were performed in triplicate and normalized to total protein content.

### Measurement of GSH

Cellular glutathione (GSH) levels were quantified using a commercial assay kit (S0053, Beyotime, China). Cells were cultured in complete medium for 24 hours before harvesting. The assay utilizes a glutathione recycling system involving 5,5′-dithiobis-(2-nitrobenzoic acid) (DTNB) and glutathione reductase. DTNB reacts with GSH to produce 2-nitro-5-thiobenzoic acid, which exhibits a yellow color detectable at 412 nm. Absorbance was measured using a microplate reader. All measurements were performed in triplicate and normalized to total protein content. Data are presented as mean ± standard deviation.

### ROS measurement

Intracellular ROS levels were detected using the fluorescent probe 2′,7′-dichlorodihydrofluorescein diacetate (DCFH-DA; Beyotime, S0033S, China). After 12 hours of culture in complete medium, cells were washed with PBS and incubated with 10 μM DCFH-DA at 37 °C for 30 minutes. Subsequently, the cells were trypsinized, resuspended in PBS, and analyzed immediately by flow cytometry. All experiments were performed in triplicate.

#### Measurement of glucose uptake assay, lactate production, and ATP levels

Glucose uptake was measured using a Glucose Uptake Assay Kit (WST-8 based; Beyotime, S0554S, China) following the manufacturer’s instructions. In brief, LUAD cells were seeded in 96-well plates at 10,000 cells per well, treated with 800 μM oleic acid for 24 hours, serum-starved overnight, and glucose-deprived in KRPH buffer. After pretreatment with test compounds or controls (e.g., insulin or phloretin), cells were incubated with 10 mM 2-deoxy-D-glucose (2-DG) for 20 minutes, washed, and lysed. Lysates were centrifuged at 14,000 × *g* for 5 minutes at 4 °C, and the resulting supernatants were reacted with detection reagent at 37 °C for 30 minutes. Absorbance was measured at 450 nm, and 2-DG6P levels were quantified using a standard curve.

Extracellular lactate levels were assessed using a Lactic Acid Content Assay Kit (Solarbio, BC2235, China). Conditioned medium was collected from cell cultures, and absorbance was read at 450 nm. Lactate concentrations were normalized to total cell count to ensure accurate comparison across samples.

Intracellular ATP content was determined with an ATP Assay Kit (Beyotime, S0027, China). Cells were lysed, and luminescence was measured at 450 nm. ATP values were normalized to cell number to account for variations in cell density. All assays were performed in at least three independent biological replicates.

### Immunohistochemistry (IHC)

A formalin-fixed paraffin-embedded (FFPE) tissue microarray (TMA) containing 55 surgically resected LUAD specimens was prepared by Servicebio (Wuhan, China). IHC was performed on sections from human LUAD samples fixed in 4% formalin and embedded in paraffin. Tissue blocks were sectioned at 4 μm thickness, mounted on slides, and incubated with primary antibodies at 4 °C overnight. After washing with PBS, the sections were incubated with an HRP-conjugated secondary antibody for 1 hour at room temperature. Signal was developed using 3,3′-diaminobenzidine (DAB; Sigma-Aldrich) for 3 minutes, followed by counterstaining with hematoxylin (Sigma-Aldrich). Two independent pathologists evaluated the stained sections based on staining intensity and the percentage of positive cells. Staining intensity was graded as follows: 0 (negative), 1 (weak), 2 (moderate), or 3 (strong). The proportion of positive cells was scored as: 0 (0%), 1 (1–24%), 2 (25–49%), 3 (50–74%), or 4 (75–100%). The following primary antibodies were used: anti-RACK1 (1:200, Proteintech, #27592-1-AP) (RRID:AB_2880917), anti-G6PD (1:400, Proteintech, #25413-1-AP) (RRID:AB_2880066), anti-LDHA (1:400, Proteintech, #19987-1-AP) (RRID:AB_10646429), anti-TRIM21 (1:400, Proteintech, #12108-1-AP) (RRID:AB_2209469), and anti-Ki67 (1:1000, Cell Signaling Technology, #9449) (RRID:AB_2797703).

### Animal studies

Male BALB/c nude mice (4 weeks old) (RRID:IMSR_GPT:D000521) were obtained from Nanjing GemPharmatech Co., Ltd. and maintained under specific pathogen-free (SPF) conditions. For the subcutaneous xenograft model, PC9 cells (1 × 10⁶) were resuspended in a 1:1 (v/v) mixture of PBS and Matrigel (BD Biosciences, #354234) and injected subcutaneously into 6-week-old male BALB/c nude mice. Tumor size was measured every three days using a caliper, and volume was calculated as follows: volume (mm³) = (width² × length) / 2. Mice were humanely euthanized when tumors in the control group reached ~500 mm³. Tumors were then excised, weighed, and prepared for further immunohistochemical analysis. All animal procedures were conducted in compliance with institutional guidelines and approved by the Institutional Animal Care and Use Committee (IACUC) of East Hospital, Tongji University (Approval No.: TJBB03724103).

### Establishment of the lung metastasis model

To establish a hematogenous pulmonary metastasis model, PC9 human lung adenocarcinoma cells (2 × 10⁶ cells in 100 µL of sterile phosphate-buffered saline [PBS]) were harvested during the logarithmic growth phase and injected into the lateral tail vein of 6-week-old male BALB/c nude mice. Two weeks post-injection, the mice were randomly assigned to treatment groups (*n* = 6 per group). Each group then received treatment with either the c-Src inhibitor Saracatinib (25 mg/kg/day) or the LDHA inhibitor Stiripentol (150 mg/kg at 3‑day intervals) for four weeks. At the experimental endpoint, all mice were humanely euthanized. The lungs were subsequently excised, and lung tissues were collected for hematoxylin and eosin (H&E) staining to assess histopathological features and metastatic tumor burden.

### Liquid chromatography-tandem mass spectrometry (LC–MS/MS)

Protein identification by LC–MS/MS was performed following an established protocol with minor modifications. In brief, 50 µg of total protein was separated by SDS-PAGE (12% gel), and relevant protein bands were excised and subjected to in-gel tryptic digestion. The resulting peptides were extracted, desalted, and reconstituted in 0.1% formic acid. LC–MS/MS analysis was carried out using a nanoflow liquid chromatography system coupled to a Q-Exactive HF mass spectrometer (Thermo Fisher Scientific). Mass spectra were acquired in data-dependent acquisition mode, and raw files were processed using MaxQuant software (version 2.1.0) for peptide and protein identification against the UniProt human database.

### Statistical analysis

All statistical analyses were performed using GraphPad Prism 8.0 (GraphPad Software, USA) (RRID:SCR_002798). Continuous variables are presented as mean ± standard deviation (SD). Comparisons of clinicopathological characteristics were conducted using unpaired Student’s *t* test (for normally distributed continuous variables) or χ² test (for categorical variables). Differences between experimental groups from in vitro and in vivo studies were evaluated using Student’s *t* test (two groups) or one-way ANOVA (multiple groups), followed by an appropriate post-hoc test. All experiments were repeated in at least three independent replicates. A p-value less than 0.05 was considered statistically significant.

## Results

### RACK1 is upregulated and associated with poor prognosis in lung adenocarcinoma (LUAD)

**Fig. 1:**
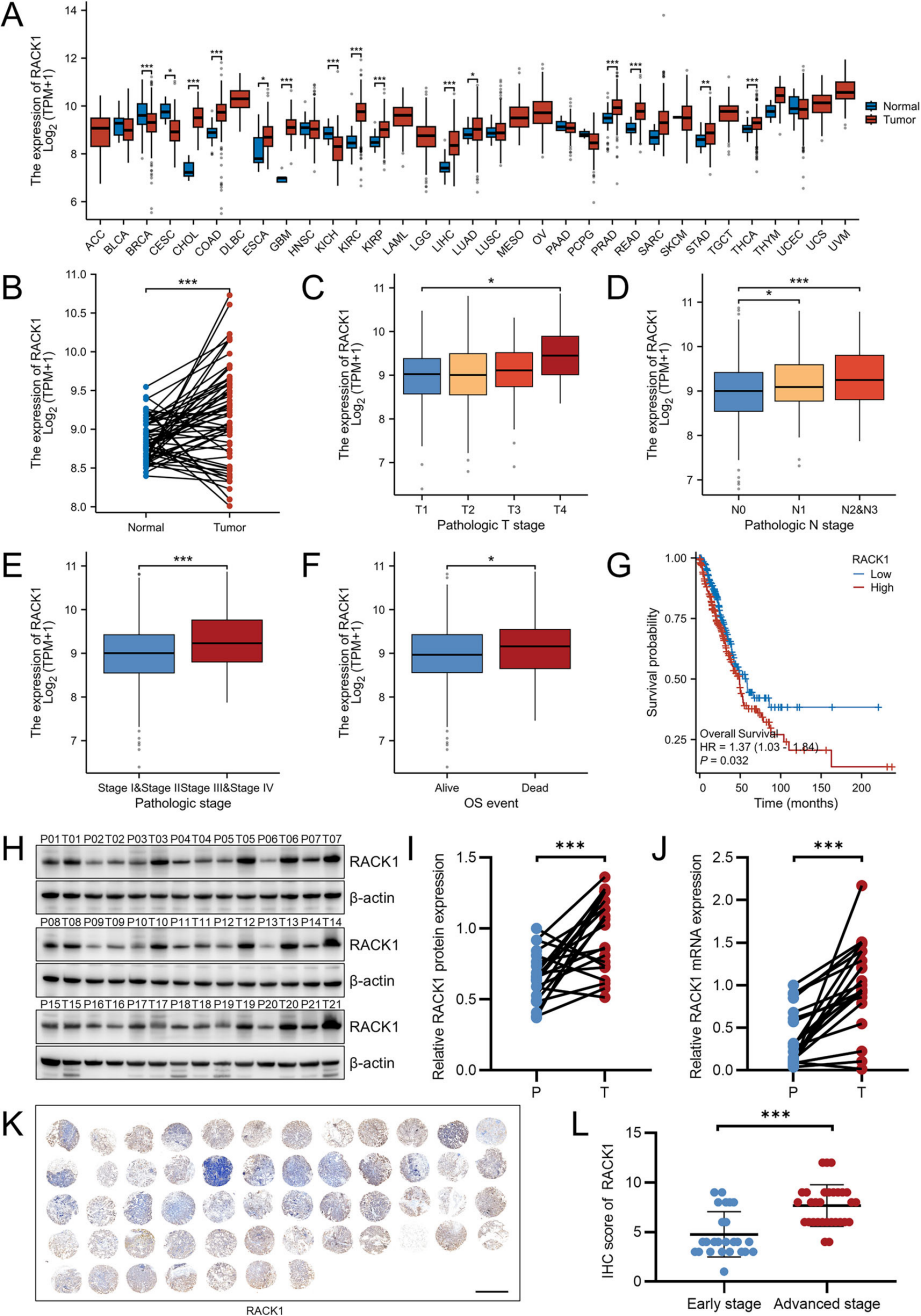
RACK1 is upregulated and associated with poor prognosis in lung adenocarcinoma (LUAD). **A** Pan-cancer analysis of RACK1 mRNA expression across human cancers from The Cancer Genome Atlas (TCGA). **B** RACK1 mRNA expression in LUAD tumors compared to matched adjacent normal lung tissues from the TCGA database. **C**–**E** RACK1 mRNA expression levels stratified by pathologic T stage (**C**), N stage (**D**), and overall pathologic stage (**E**) in the TCGA LUAD cohort. **F** RACK1 mRNA expression levels in LUAD patients who were alive versus those who had died. **G** Kaplan–Meier overall survival analysis of LUAD patients stratified by high vs. low RACK1 mRNA expression. **H** Representative western blot images of RACK1 protein expression in paired LUAD tumors (**T**) and paracancerous tissue (**P**). **I** Quantitative analysis of RACK1 protein expression from (**H**). **J** RACK1 mRNA expression levels in clinical LUAD samples as determined by RT-qPCR. **K** Representative images of RACK1 immunohistochemical (IHC) staining on a tissue microarray (TMA) containing 55 LUAD cases. Scale bar = 2000 µm (**L**) Quantitative IHC scores of RACK1 expression in early-stage vs. advanced-stage LUAD based on TMA analysis. Error bars represent mean ± S.D. *P < 0.05, ***P < 0.001.

To investigate the expression profile of RACK1 in lung adenocarcinoma (LUAD), we first conducted an in silico analysis of data from The Cancer Genome Atlas (TCGA). As shown in Fig. 1A, RACK1 expression was significantly upregulated in multiple solid tumors, including LUAD, glioblastoma (GBM), and prostate adenocarcinoma (PRAD), compared to adjacent normal tissues (Fig. 1A, B). Further analysis revealed that RACK1 expression levels correlated with disease progression, showing a gradual increase with advancing pathologic T and N stages, and were higher in patients with advanced pathologic stage (Fig. 1C–E). Survival analysis indicated that LUAD patients with high RACK1 expression had shorter overall survival compared to those with low expression (Fig. 1F, G). Consistent with the bioinformatics findings, immunoblotting confirmed elevated RACK1 protein levels in 21 clinical LUAD specimens compared to their paired adjacent normal tissues (Fig. 1H, I). This upregulation was further validated at the transcriptional level using quantitative real-time PCR (qRT-PCR) (Fig. 1J). Moreover, immunohistochemical analysis of a tissue microarray (TMA) containing 55 LUAD cases demonstrated higher RACK1 protein expression in advanced-stage tumors compared to early-stage disease (Fig. 1K, L). Taken together, these data indicate that RACK1 is upregulated in LUAD and that its elevated expression is associated with tumor progression and poor prognosis.

### RACK1 overexpression enhances the proliferation and invasion of LUAD cells

**Fig. 2:**
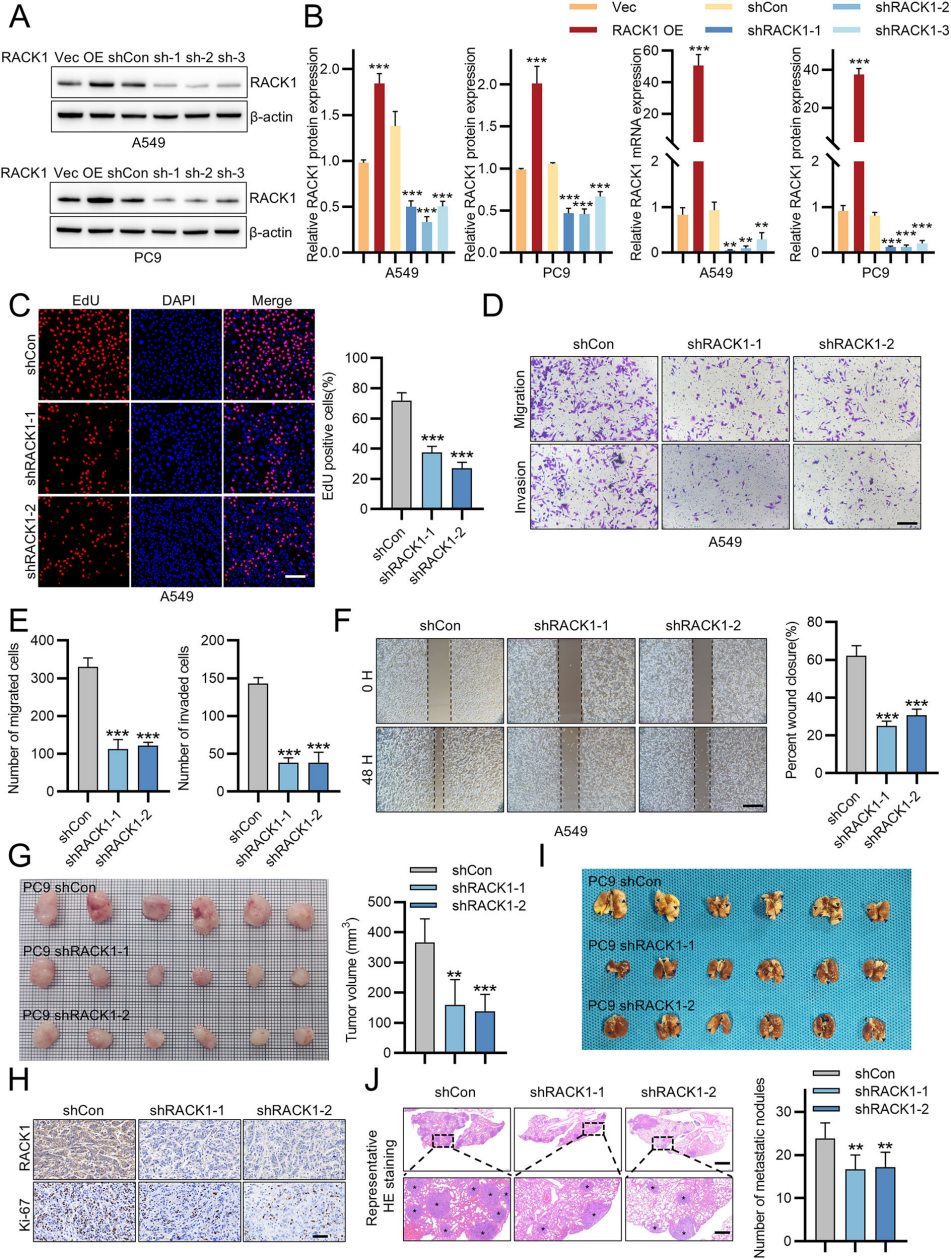
RACK1 overexpression enhances the proliferation and invasion of LUAD cells. **A** Western blot analysis of RACK1 protein levels in A549 and PC9 cells following stable overexpression or knockdown, with β-actin serving as a loading control. **B** Quantification of RACK1 expression from western blot (protein) and RT-qPCR (mRNA) analyses. **C** Representative images (left) and quantification (right) of EdU incorporation assays in A549 cells following stable knockdown or control, indicating cell proliferation. Scale bar = 50 µm **D** Representative images of transwell migration (upper chamber) and invasion (Matrigel-coated chamber) assays in A549 cells following stable knockdown or control. Scale bar = 100 µm. **E** Quantitative analysis of migrated and invaded cells from (**D**). **F** Representative images (left) and quantification (right) of wound-healing assays in A549 cells following stable knockdown or control. Scale bar = 100 µm. **G** Representative images of xenograft tumors (left) and quantitative analysis of tumor volume (right) from PC9 control and RACK1-knockdown cells in nude mice (*n* = 6). **H** Immunohistochemical (IHC) staining of RACK1 and the proliferation marker Ki-67 in representative xenograft tumor sections. Scale bar = 20 µm. **I** Representative images of lung metastases in mice following tail vein injection of control or RACK1-knockdown PC9 cells. Metastatic lesions are labelled. **J** H&E staining of lung sections from the metastasis model, with representative images indicating metastatic foci. Scale bar = 500 µm and 100 µm. Each experiment was performed in three independent biological replicates (*n* = 3). Error bars represent mean ± S.D. **P < 0.01, ***P < 0.001.

To investigate the functional role of RACK1 in LUAD, we transduced A549 and PC9 LUAD cell lines with lentiviral vectors to achieve RACK1 overexpression or knockdown (shRACK1). The efficiency of these manipulations was confirmed by immunoblotting and qRT-PCR (Fig. 2A, B). Among several shRNA constructs, shRACK1-1 and shRACK1-2 demonstrated the most potent suppression of RACK1 and were thus selected for subsequent experiments. EdU assay results revealed that RACK1 knockdown significantly suppressed the proliferation of LUAD cells (Fig. 2C and Supplementary Fig. S1A). Furthermore, transwell invasion, migration, and wound-healing assays demonstrated that RACK1 depletion also markedly impaired the invasive and migratory capacities of these cells (Fig. 2D–F and Supplementary Fig. S1B, C). These in vitro findings were corroborated in vivo. In a subcutaneous xenograft model, tumors with RACK1 knockdown exhibited significantly slower growth rates compared to those in the vector control group (Fig. 2G). Immunohistochemical analysis of these tumors revealed decreased Ki-67 positivity following RACK1 depletion (Fig. 2H). Moreover, in a lung metastasis model, RACK1 knockdown profoundly impeded the metastatic potential of LUAD cells (Fig. 2I, J). Taken together, these data indicate that RACK1 promotes proliferation, invasion, and metastasis in LUAD.

### RACK1 facilitates glycolysis and the pentose phosphate pathway in LUAD

**Fig. 3:**
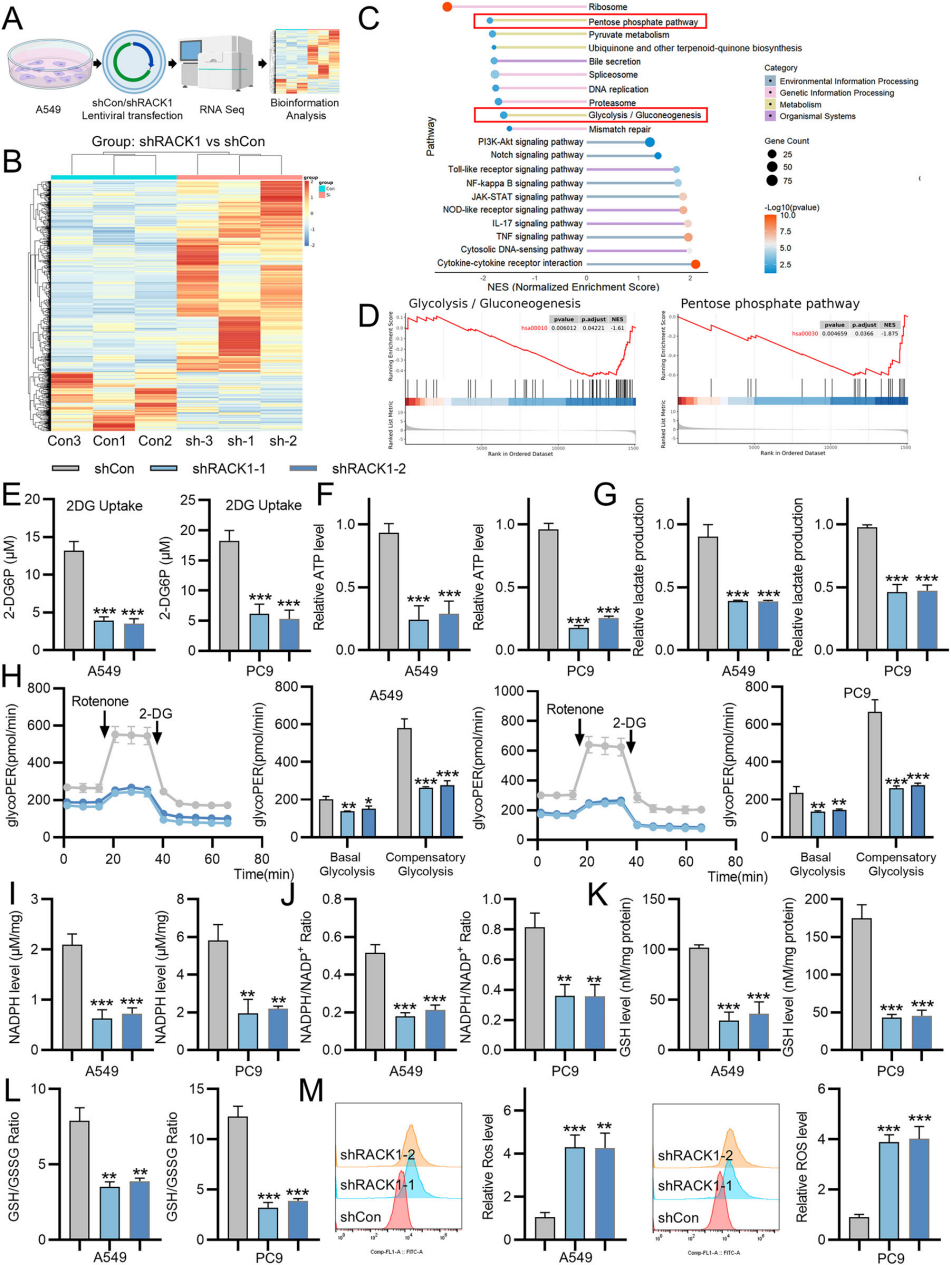
RACK1 facilitates glycolysis and the pentose phosphate pathway in LUAD. **A** Schematic diagram of the RNA sequencing (RNA-seq) strategy using control and RACK1-knockdown A549 cells. **B** Heatmap of differentially expressed genes between control and RACK1-knockdown A549 cells. **C** Gene Set Enrichment Analysis (GSEA) showing upregulated and downregulated pathways upon RACK1 knockdown. **D** Gene Set Enrichment Analysis (GSEA) plots showing significant negative enrichment of the “Glycolysis” and “Pentose Phosphate Pathway” gene sets in RACK1-knockdown cells. **E**–**G** Functional assessment of glycolytic activity, including glucose consumption (**E**), intracellular ATP levels (**F**), and lactate production (**G**) in control and RACK1-knockdown LUAD cells. **H** GlycoPER assay quantifying glycolytic activity in control and RACK1-knockdown LUAD cells. **I**–**L** Measurement of pentose phosphate pathway metabolites and redox status, including intracellular NADPH levels (**I**), NADPH/NADP+ ratio (**J**), GSH levels (**K**), and GSH/GSSG ratio (**L**) in control and RACK1-knockdown LUAD cells (*n* = 3). **M** Representative flow cytometry histograms (left) and quantitative analysis (right) of intracellular ROS levels in control and RACK1-knockdown LUAD cells. Each experiment was performed in three independent biological replicates (*n* = 3). Error bars represent mean ± S.D. *P < 0.05, **P < 0.01, ***P < 0.001.

To elucidate the mechanisms by which RACK1 promotes LUAD progression, we performed RNA sequencing (RNA-Seq) on control and RACK1-knockdown A549 cells (Fig. 3A, B). Gene Set Enrichment Analysis (GSEA) revealed that RACK1 depletion significantly impaired several key oncogenic pathways, including the PI3K-AKT, NF-κB signaling pathway and pathways related to cell viability (Fig. 3C). Furthermore, GSEA indicated a significant suppression of metabolic pathways, notably glycolysis and the pentose phosphate pathway (PPP) (Fig. 3D) [22, 23]. To directly assess whether RACK1 regulates glycolysis in LUAD, we measured key parameters of cellular energy metabolism. RACK1 knockdown reduced glucose consumption, ATP levels, and lactate production in LUAD cells (Fig. 3E–G). Consistent with this, GlycoPER measurements demonstrated that RACK1 depletion suppressed both basal glycolysis and compensatory glycolysis (Fig. 3H). We next investigated the impact of RACK1 on the PPP. In RACK1-knockdown cells, intracellular NADPH levels and the NADPH/NADP⁺ ratio were significantly decreased (Fig. 3I, J), accompanied by reduced GSH levels and GSH/GSSG ratio (Fig. 3K, L). Consistent with impaired antioxidant capacity, reactive oxygen species (ROS) levels were markedly elevated following RACK1 knockdown (Fig. 3M). Taken together, these results indicate that RACK1 is required for maintaining both glycolytic flux and PPP function in LUAD cells.

### RACK1 promotes the pentose phosphate pathway by mediating c-Src-dependent phosphorylation of G6PD

**Fig. 4:**
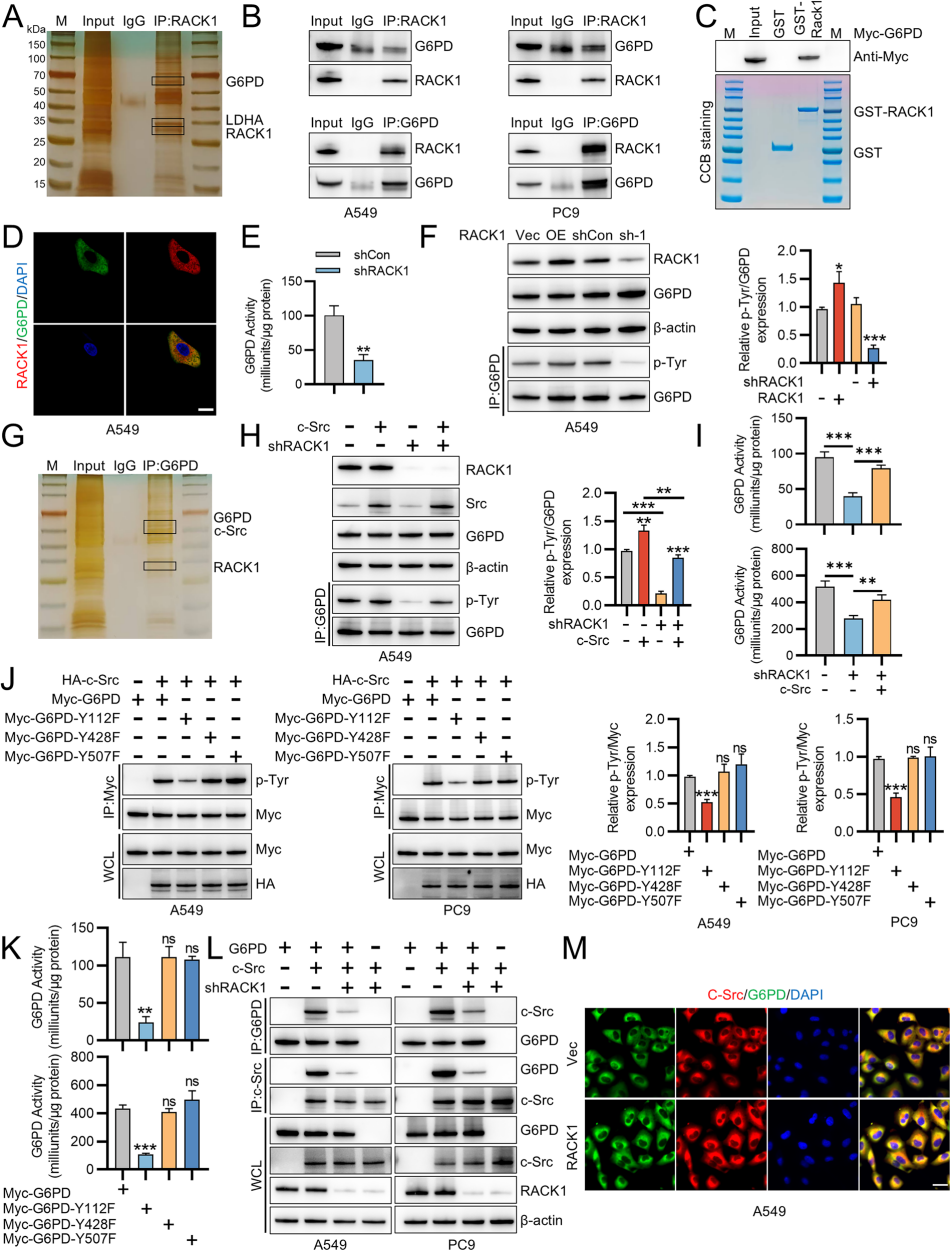
RACK1 scaffolds c-Src to phosphorylate and activate G6PD. **A** Silver staining of proteins co-immunoprecipitated (Co-IP) with RACK1 from A549 cell lysates, identifying G6PD and LDHA as specific binding partners. **B** Endogenous interaction between RACK1 and G6PD confirmed by reciprocal Co-IP in LUAD cells. **C** GST pull-down assay demonstrating a direct physical interaction between purified recombinant RACK1 and G6PD proteins. **D** Immunofluorescence (IF) images showing co-localization of RACK1 (red) and G6PD (green) in LUAD cells. Scale bar = 10 µm. **E** G6PD enzymatic activity assay in control versus RACK1-knockdown A549 cells. **F** Western blot analysis of G6PD tyrosine phosphorylation (p-Tyr) in A549 cells with modulated RACK1 expression, with quantification on the right. **G** Silver staining of proteins co-immunoprecipitated with G6PD from A549 cell lysates, identifying c-Src as an interacting kinase. **H** Western blot analysis showing that the reduction in G6PD tyrosine phosphorylation upon RACK1 knockdown is rescued by c-Src overexpression, with quantification on the right. **I** G6PD enzymatic activity upon RACK1 knockdown and subsequent c-Src re-expression. **J** Tyrosine phosphorylation levels of wild-type (WT) G6PD and its point mutants (Y112F, Y428F, Y507F) when co-expressed with c-Src, with quantification on the right. **K** G6PD enzymatic activity in G6PD-knockout LUAD cells reconstituted with G6PD-WT or the indicated tyrosine mutants. **L** Co-IP assays demonstrating that RACK1 knockdown impairs the binding between c-Src and G6PD. **M** IF images showing enhanced co-localization of G6PD (green) and c-Src (red) in RACK1-overexpressing A549 cells. Scale bar = 20 µm. Error bars represent mean ± S.D. *P < 0.05, **P < 0.01, ***P < 0.001.

To elucidate the mechanism by which RACK1 promotes the PPP, we performed co-immunoprecipitation (Co-IP) assays in A549 cells to identify its binding partners. Silver staining and subsequent mass spectrometry analysis revealed G6PD, the first and rate-limiting enzyme of the PPP, as a top candidate (Fig. 4A and Supplementary Fig. S2A). The endogenous interaction between RACK1 and G6PD was confirmed by Co-IP in LUAD cells (Fig. 4B), and a direct physical interaction was demonstrated using a GST pull-down assay with purified proteins (Fig. 4C). Immunofluorescence (IF) analysis further showed co-localization of RACK1 and G6PD in LUAD cells (Fig. 4D and Supplementary Fig. S2B). Functionally, RACK1 knockdown significantly suppressed G6PD enzymatic activity (Fig. 4E and Supplementary Fig. S2C). Given that G6PD activity is known to be regulated by post-translational modifications, including phosphorylation [24, 25], we examined the effect of RACK1 on G6PD phosphorylation. RACK1 depletion decreased tyrosine phosphorylation of G6PD, while RACK1 overexpression increased it (Fig. 4F and Supplementary Fig. S2D). As RACK1 is a scaffold protein known to modulate activated protein kinase C (PKC), such as c-Src [26], we hypothesized that it might facilitate G6PD phosphorylation via c-Src. Indeed, Co-IP assays identified c-Src as a G6PD-interacting kinase (Fig. 4G and Supplementary Fig. S2E). Crucially, the reduction in G6PD phosphorylation and activity upon RACK1 knockdown was effectively rescued by the re-expression of c-Src (Fig. 4H, I and Supplementary Fig. S2F). To identify the specific phosphorylation site, we generated point mutations at conserved tyrosine residues. Mutation of Tyr112 (Y112F), but not Tyr428 or Tyr507, abolished c-Src-induced G6PD phosphorylation and impaired G6PD activity, identifying Tyr112 as the primary c-Src phosphorylation site (Fig. 4J, K). We next examined whether RACK1 facilitates the c-Src-G6PD interaction. Co-IP assays showed that RACK1 knockdown impaired the association between c-Src and G6PD (Fig. 4L), and IF analysis revealed enhanced co-localization of these two proteins upon RACK1 overexpression (Fig. 4M and Supplementary Fig. S2G). Together, these data indicate that RACK1 scaffolds c-Src to phosphorylate G6PD at Tyr112, thereby enhancing G6PD activity and promoting PPP flux.

### RACK1 promotes glycolysis by enhancing LDHA protein stability

**Fig. 5:**
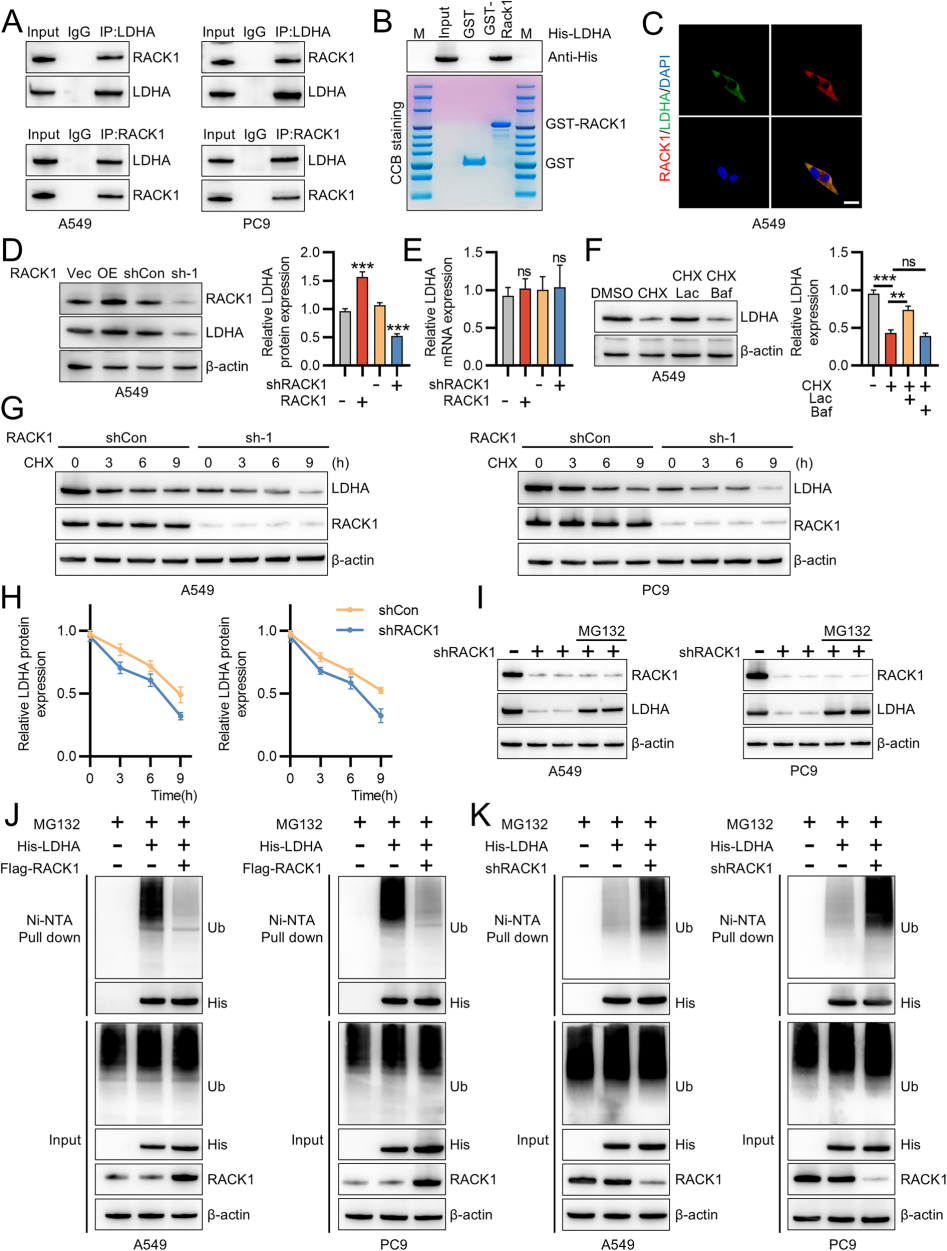
RACK1 promotes glycolysis by enhancing LDHA protein stability. **A** Endogenous interaction between RACK1 and LDHA confirmed by reciprocal co-immunoprecipitation (Co-IP) in LUAD cells. **B** GST pull-down assay demonstrating a direct physical interaction between purified recombinant RACK1 and LDHA proteins. **C** Immunofluorescence (IF) images showing co-localization of RACK1 (red) and LDHA (green) in LUAD cells. Scale bar = 20 µm. **D** Western blot analysis (left) and quantification (right) of LDHA protein levels in A549 cells with RACK1 knockdown or overexpression. **E** RT-qPCR analysis of LDHA mRNA levels in A549 cells with modulated RACK1 expression. **F** Western blot analysis of LDHA protein levels in A549 cells treated for 9 hours with the protein synthesis inhibitor cycloheximide (CHX, 4 µg/mL) in combination with the proteasome inhibitor Lactacystin (Lac, 20 µM) or the lysosome inhibitor bafilomycin A1 (Baf, 200 nM). DMSO was used as a vehicle control. Quantification is shown on the right. **G** Western blot analysis of LDHA protein levels in control (shCon) or RACK1-knockdown LUAD cells treated with CHX and harvested at the indicated time points. **H** Quantification of LDHA protein half-life from (**G**) in A549 (left) and PC9 (right) cells. **I** Western blot analysis of LDHA protein levels in RACK1-knockdown LUAD cells treated with or without the proteasome inhibitor MG132 (20 µM) for 9 hours. **J**, **K** Ubiquitination assays in LUAD cells stably expressing His-tagged LDHA. Cells with RACK1 overexpression (**J**) or knockdown (**K**) were treated with MG132 (20 µM) for 9 hours before harvest. His-LDHA was purified using Ni-NTA pull-down and immunoblotted with an anti-ubiquitin antibody. Data are presented as the mean ± s.e.m. (*n* = 3 biologically independent samples). Statistical significance was determined by a two-tailed unpaired Student’s *t* test. Error bars represent mean ± S.D. **P < 0.01, ***P < 0.001.

Our prior mass spectrometry analysis also identified LDHA—a crucial enzyme that catalyzes the final step of aerobic glycolysis—as a potential RACK1-interacting protein (Fig. 4A and Supplementary Fig. S3A). The endogenous interaction between RACK1 and LDHA was confirmed by reciprocal Co-IP in LUAD cells (Fig. 5A), and a direct physical interaction was demonstrated by GST pull-down assay using purified proteins (Fig. 5B). Immunofluorescence analysis further showed co-localization of RACK1 and LDHA in LUAD cells (Fig. 5C and Supplementary Fig. S3B). To determine whether RACK1 regulates glycolysis through LDHA, we modulated RACK1 expression in LUAD cells. RACK1 knockdown reduced LDHA protein levels, while its overexpression increased them (Fig. 5D and Supplementary Fig. S3C). Notably, LDHA mRNA levels remained unchanged, suggesting that RACK1 regulates LDHA at the post-translational level (Fig. 5E and Supplementary Fig. S3D). We therefore investigated LDHA protein stability. Treatment with the protein synthesis inhibitor cycloheximide (CHX) led to a rapid decrease in LDHA levels, which was blocked by the proteasome inhibitor Lactacystin but not by the lysosome inhibitor Bafilomycin A1 (Fig. 5F and Supplementary Fig. S3E). RACK1 knockdown shortened the half-life of LDHA, and this effect was reversed by the proteasome inhibitor MG132 (Fig. 5G-I). To directly test whether RACK1 inhibits the ubiquitin-proteasomal degradation of LDHA, we assessed LDHA ubiquitination levels. RACK1 overexpression reduced LDHA ubiquitination, whereas RACK1 knockdown increased it (Fig. 5J, K). Taken together, these data indicate that RACK1 binds to LDHA and enhances its stability by suppressing ubiquitin-proteasomal degradation.

### RACK1 and TRIM21 competitively bind to LDHA to regulate its protein stability

**Fig. 6:**
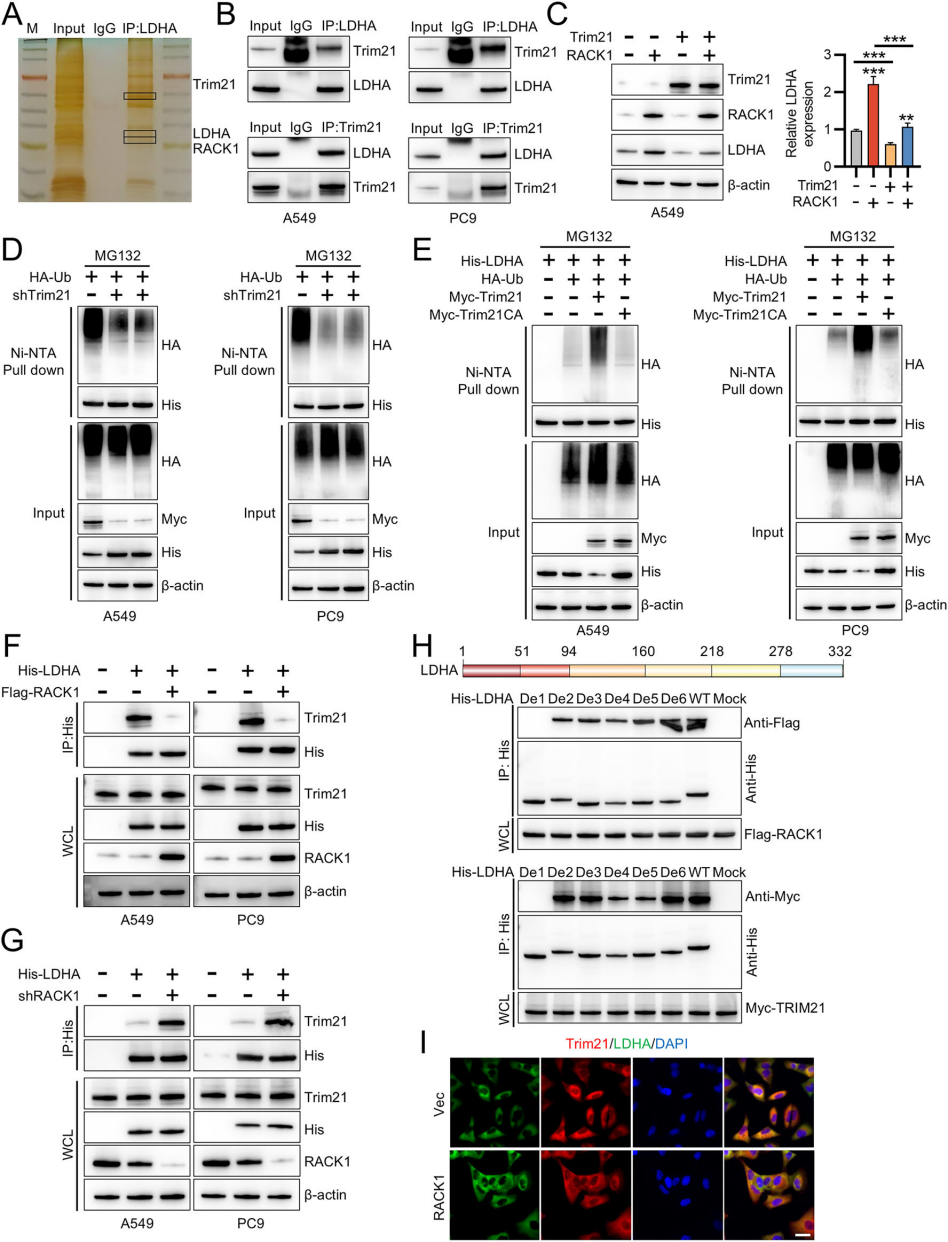
RACK1 and TRIM21 competitively bind to LDHA to regulate its protein stability. **A** Silver staining of proteins co-immunoprecipitated (Co-IP) with LDHA from A549 cell lysates, identifying RACK1 and TRIM21 as specific binding partners. **B** Endogenous interaction between TRIM21 and LDHA confirmed by reciprocal Co-IP in LUAD cells. **C** Western blot analysis (left) and quantification (right) of LDHA protein levels in A549 cells with TRIM21 overexpression and RACK1 overexpression. **D** Ubiquitination assay in LUAD cells stably expressing His-LDHA. Cells with TRIM21 knockdown were treated with MG132 (20 µM) for 9 hours before His-LDHA was purified by Ni-NTA pull-down and immunoblotted with an anti-ubiquitin antibody. **E** Ubiquitination assay in LUAD cells expressing wild TRIM21 or the catalytically inactive mutant TRIM21-CA (C16A/C31A/H33W), showing that TRIM21’s E3 ligase activity is required for LDHA ubiquitination. **F**, **G** Co-IP assays demonstrating that RACK1 overexpression (**F**) or knockdown (**G**) impairs the binding between TRIM21 and LDHA. **H** Domain-mapping analysis. Schematic of LDHA wild-type (WT) and deletion mutants (Upper). Co-IP assays in 293 T cells showing that both RACK1 and TRIM21 bind to the N-terminal region (amino acids 1-51) of LDHA. **I** Immunofluorescence (IF) images showing reduced co-localization of LDHA (green) and TRIM21 (red) in RACK1-overexpressed A549 cells. Scale bar = 10 µm. Data are presented as the mean ± s.e.m. (*n* = 3 biologically independent samples). Statistical significance was determined by a two-tailed unpaired Student’s *t* test. Error bars represent mean ± S.D. ***P < 0.001.

To elucidate how RACK1 enhances LDHA stability, we performed co-immunoprecipitation (Co-IP) coupled with mass spectrometry, which identified the E3 ubiquitin ligase TRIM21 as a top candidate (Fig. 6A and Supplementary Fig. S4A). We confirmed the endogenous interaction between TRIM21 and LDHA in LUAD cells by reciprocal Co-IP (Fig. 6B). Functionally, TRIM21 overexpression markedly reduced LDHA protein levels, and this reduction was rescued by co-expression of RACK1 (Fig. 6C and Supplementary Fig. S4B). Conversely, TRIM21 knockdown decreased LDHA ubiquitination, whereas overexpression of wild-type TRIM21, but not its catalytically inactive mutant TRIM21CA (TRIM21-C16A/C31A/H33W) [27], enhanced it (Fig. 6D, E). We next examined whether RACK1 and TRIM21 compete for LDHA binding. RACK1 overexpression diminished the TRIM21-LDHA interaction, while RACK1 knockdown enhanced it (Fig. 6F, G). To define the molecular basis for this competition, we generated truncated LDHA constructs. Domain-mapping analyses localized the shared binding site for both RACK1 and TRIM21 to the LDHA N-terminal region (amino acids 1-51) (Fig. 6H). Immunofluorescence analysis further showed that RACK1 overexpression decreased the co-localization of TRIM21 and LDHA (Fig. 6I and Supplementary Fig. S4C). Together, these data indicate that RACK1 and TRIM21 competitively bind to the N-terminal domain of LDHA, with RACK1 binding protecting LDHA from TRIM21-mediated ubiquitination and degradation.

### RACK1 promotes LUAD malignancy by coordinately enhancing glycolysis via LDHA and the pentose phosphate pathway via G6PD

**Fig. 7:**
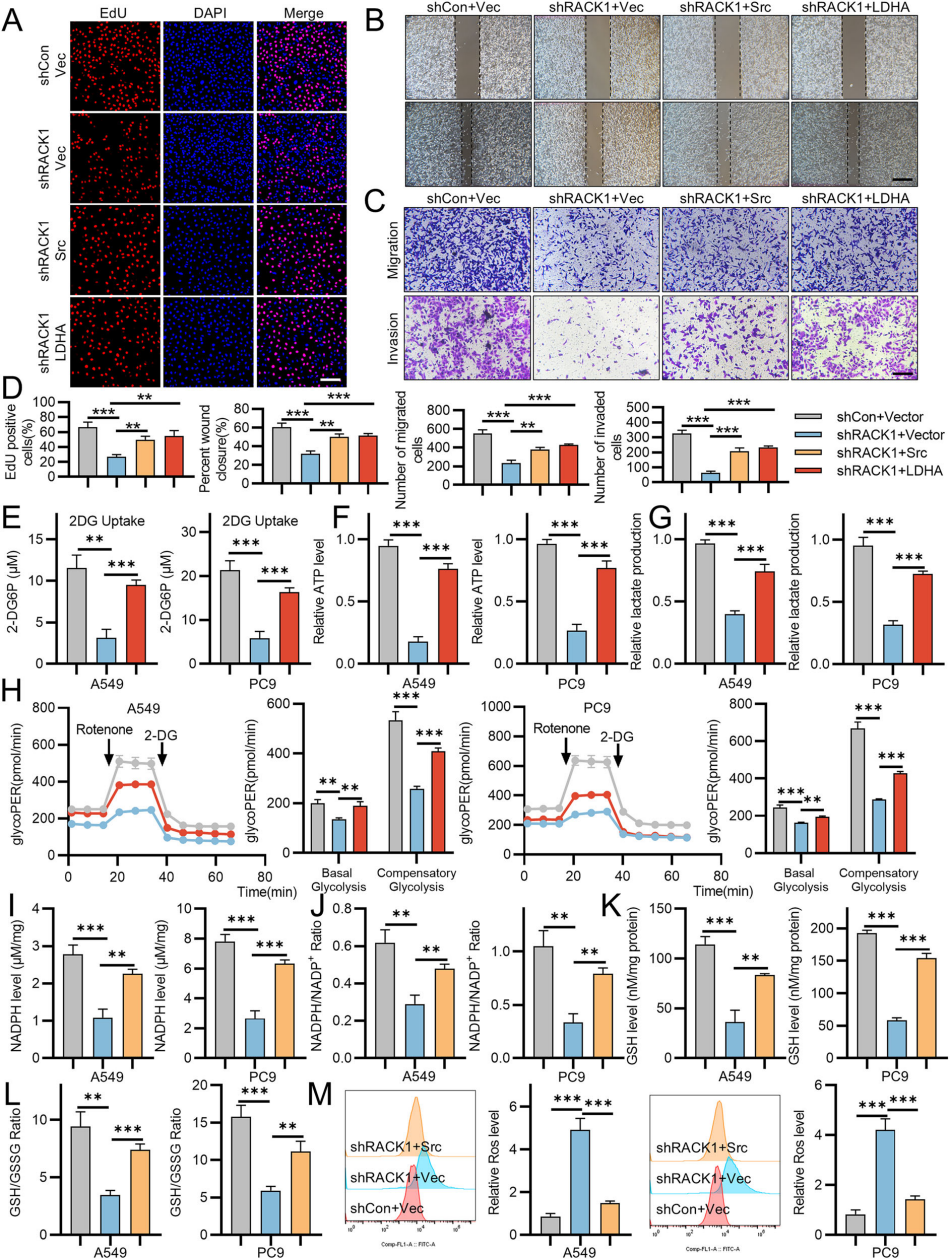
RACK1 promotes LUAD malignancy by coordinately enhancing glycolysis via LDHA and the pentose phosphate pathway via G6PD. **A** Representative images (upper panel) and quantification (lower panel) of EdU incorporation assays in control and RACK1-knockdown A549 cells, showing that the proliferation deficit is rescued by re-expression of c-Src or LDHA. Scale bar = 50 µm. **B** Representative images (upper panel) and quantification (lower panel) of wound-healing assays in control and RACK1-knockdown A549 cells, showing that the migration defect is rescued by re-expression of c-Src or LDHA. Scale bar = 100 µm. **C** Representative images of transwell migration (upper chamber) and invasion (Matrigel-coated chamber) assays in control and RACK1-knockdown A549 cells, showing that the invasive and migratory deficits are rescued by re-expression of c-Src or LDHA. Scale bar = 100 µm. **D** Quantitative analysis of the EdU incorporation, wound-healing assays, transwell migration and invasion assays from (**A**–**C**). **E**–**G** Functional assessment of glycolytic activity, including glucose consumption (**E**), intracellular ATP levels (**F**), and lactate production (**G**) in control and RACK1-knockdown LUAD cells, showing that the glycolytic impairment is specifically rescued by LDHA re-expression. **H** GlycoPER assay quantifying glycolytic capacity in control and RACK1-knockdown LUAD cells, showing rescue by LDHA re-expression. **I**–**L** Measurement of pentose phosphate pathway metabolites and redox status, including intracellular NADPH levels (**I**), NADPH/NADP+ ratio (**J**), GSH levels (**K**), and GSH/GSSG ratio (**L**) in control and RACK1-knockdown LUAD cells (*n* = 3), showing that the PPP and redox impairments are specifically rescued by c-Src re-expression. **M** Representative flow cytometry histograms (left) and quantitative analysis (right) of intracellular ROS levels in control and RACK1-knockdown LUAD cells, showing that elevated ROS is specifically normalized by c-Src re-expression. Data are presented as the mean ± s.e.m. (*n* = 3 biologically independent samples). Statistical significance was determined by a two-tailed unpaired Student’s *t* test. **P < 0.01, ***P < 0.001.

To definitively establish the roles of G6PD and LDHA in RACK1-mediated LUAD progression, we performed a series of rescue experiments. EdU assays demonstrated that the proliferation deficit induced by RACK1 knockdown was effectively reversed by the re-expression of either c-Src or LDHA (Fig. 7A and Supplementary Fig. S5A). Similarly, in transwell invasion, migration, and wound-healing assays, the impaired invasive and migratory capacities resulting from RACK1 depletion were substantially restored by c-Src or LDHA overexpression (Fig. 7B–D and Supplementary Fig. S5B, C). We next examined whether these phenotypic rescues were accompanied by restoration of the underlying metabolic pathways. The decreased glycolytic activity—including reduced glucose consumption, ATP levels, and lactate production—in RACK1-knockdown cells was specifically reversed by LDHA re-expression (Fig. 7E–G). GlycoPER measurements confirmed that LDHA re-expression restored glycolytic capacity in RACK1-depleted cells (Fig. 7H). Conversely, the metabolic perturbations in the PPP—manifested as altered NADPH/GSH levels and elevated ROS—caused by RACK1 knockdown were specifically counteracted by c-Src re-expression, which restored redox homeostasis (Fig. 7I–M). Taken together, these data indicate that RACK1 promotes LUAD malignancy through two parallel pathways: LDHA-mediated glycolysis and c-Src-mediated PPP activation.

### Targeting the RACK1-c-Src-G6PD and RACK1-LDHA axes as a therapeutic strategy against LUAD

**Fig. 8:**
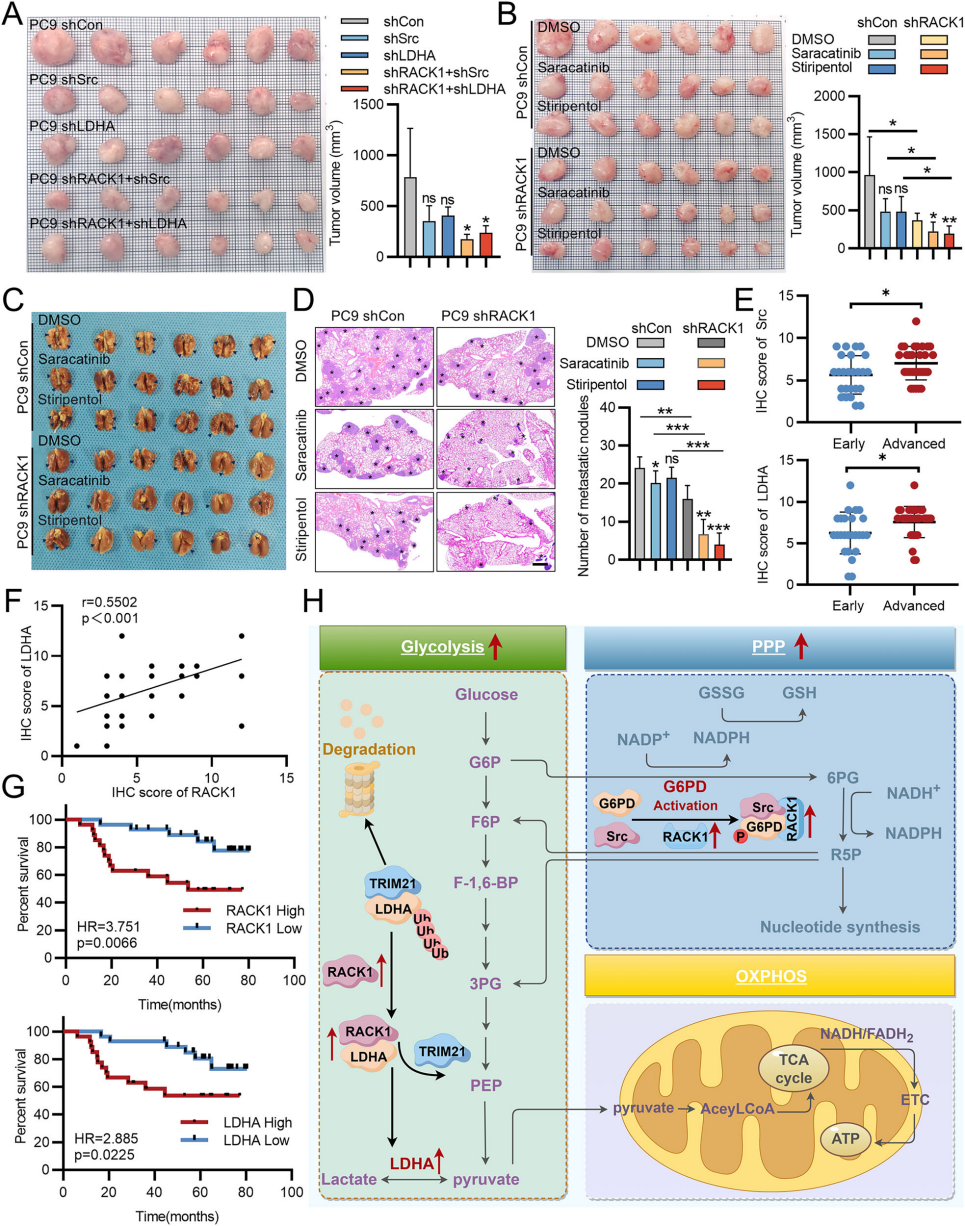
Targeting the RACK1-c-Src-G6PD and RACK1-LDHA Axes as a Therapeutic Strategy Against LUAD. **A** Representative images (left) and quantitative analysis of tumor volume (right) of xenograft tumors from PC9 cells (1 × 10⁶ cells per mouse) with single or combined knockdown of RACK1, c-Src, or LDHA after 4 weeks (n = 6). **B** Representative images (left) and quantitative analysis of tumor volume (right) of xenograft tumors from PC9 cells treated with the c-Src inhibitor Saracatinib (25 mg/kg/day) or the LDHA inhibitor Stiripentol (150 mg/kg at 3‑day intervals), alone or in combination with RACK1 knockdown, for 4 weeks (n = 6). **C** Representative images of lung metastases in mice following tail vein injection of PC9 cells (2×10⁶ cells per mouse) with the indicated treatments (as in **B**) for 4 weeks (n = 6). **D** H&E staining of lung sections from the metastasis model in (**C**), with indicating metastatic foci. Scale bar = 500 µm. **E** Quantitative IHC scores of c-Src (upper) and LDHA (lower) expression in early-stage versus advanced-stage LUAD patient samples from a tissue microarray (TMA). **F** Scatter plot showing a positive correlation between RACK1 and LDHA protein expression in LUAD patient samples (linear regression, *R* = 0.5502, *P* < 0.001). **G** Kaplan–Meier curves for progression-free survival of LUAD patients stratified by high or low expression of RACK1 (upper) and LDHA (lower). **H** Schematic model summarizing the role of RACK1 in promoting LUAD progression by co-activating glycolysis via the LDHA axis and the pentose phosphate pathway via the c-Src-G6PD axis. Statistical significance was determined by a two-tailed unpaired Student’s *t* test (**A**, **B**, **E**) or Log-rank test (**G**). *P < 0.05, **P < 0.01, ***P < 0.001.

To evaluate the therapeutic potential of disrupting the RACK1-driven metabolic axes in vivo, we established LUAD xenograft models in BALB/c mice. Individual knockdown of c-Src or LDHA alone had minimal effect on tumor growth, whereas combination strategies targeting both RACK1 and c-Src, or both RACK1 and LDHA, significantly suppressed tumor progression (Fig. 8A). We next employed pharmacological inhibitors: Saracatinib, a preclinical c-Src kinase inhibitor [28], and Stiripentol, a documented LDHA inhibitor in clinical trials [29]. Consistent with the genetic data, single-agent treatment showed limited anti-tumor effects, whereas combining either inhibitor with RACK1 knockdown resulted in pronounced tumor growth suppression (Fig. 8B). This enhanced efficacy was further confirmed in a lung metastasis model, wherein the combination strategies markedly inhibited metastatic burden (Fig. 8C, D). Clinical correlation analysis reinforced the relevance of these axes. Both c-Src and LDHA were highly expressed in advanced-stage LUAD patients (Fig. 8E and Supplementary Fig. S6A). Furthermore, LDHA protein levels exhibited a positive correlation with RACK1 expression, and patients with high expression of RACK1 or LDHA suffered from significantly poorer overall survival (Fig. 8F, G). Taken together, these data indicate that the RACK1-c-Src-G6PD and RACK1-LDHA axes contribute to metabolic reprogramming and tumor progression in LUAD, and that co-targeting these pathways in combination with RACK1 represents a potential therapeutic strategy.

## Discussion

Lung adenocarcinoma (LUAD) remains a leading cause of cancer mortality, with a 5-year survival rate of only 15% despite therapeutic advances [30, 31]. This poor outcome is largely attributable to late diagnosis and therapeutic resistance, challenges exacerbated by pronounced intra- and inter-tumor heterogeneity [32, 33]. Identifying key molecular drivers that underlie LUAD progression is therefore urgently needed. Here, we identify RACK1 as a critical regulator of LUAD pathogenesis, demonstrating that its elevated expression correlates with advanced disease and poor prognosis. Mechanistically, RACK1 drives metabolic reprogramming through dual pathways: stabilizing LDHA to enhance glycolysis and scaffolding c-Src to activate G6PD, thereby promoting PPP flux.

RACK1, a WD-repeat protein family member originally identified as a scaffold for activated PKC, is now recognized as a multifaceted regulator of diverse biological processes, including neural development, cell migration, and angiogenesis [34–36]. It exerts its functions by interacting with various partners to facilitate protein shuttling, modulate activity, alter intermolecular interactions, or regulate protein stability [37, 38]. While RACK1 has been implicated in LUAD progression, its underlying mechanisms remain largely unexplored. Through mass spectrometry and functional studies, we now delineate its role in co-activating two pivotal metabolic pathways: glycolysis and the pentose phosphate pathway (PPP). Specifically, RACK1 promotes glycolysis by stabilizing LDHA, and enhances PPP flux by facilitating the c-Src-mediated phosphorylation of G6PD. Targeting RACK1 effectively suppressed tumor growth in preclinical models. However, the absence of potent RACK1-specific inhibitors has hampered its translational development, prompting our investigation into alternative therapeutic strategies.

G6PD, the rate-limiting enzyme of the PPP, generates NADPH to support lipid synthesis and maintain redox homeostasis [39]. G6PD is frequently upregulated in LUAD, bolstering malignant phenotypes such as apoptosis resistance and invasion [40, 41]; however, the regulation of its activity is not fully understood. Beyond transcriptional control, post-translational modifications (PTMs) including phosphorylation critically regulate G6PD [25]. Here, we demonstrate that RACK1 scaffolds c-Src to phosphorylate G6PD at Tyr112, thereby enhancing its activity. This adds to the growing understanding of post-translational regulation of metabolic enzymes and identifies a specific phosphorylation site critical for G6PD function in LUAD. Concurrently, we uncovered a non-canonical mechanism of LDHA regulation. LDHA, the enzyme catalyzing the final step of glycolysis, is frequently upregulated in cancer [42]. We show that RACK1 stabilizes LDHA by competing with the E3 ligase TRIM21 for binding to its N-terminal domain, thereby protecting LDHA from ubiquitin-proteasomal degradation. This competitive binding model represents a novel paradigm for regulating glycolytic enzyme stability, distinct from previously described mechanisms involving direct phosphorylation or acetylation [43, 44].

The therapeutic implications of our findings are noteworthy. Given the absence of clinical RACK1 inhibitors, we explored alternative strategies. Genetic or pharmacological inhibition of the downstream effectors c-Src (with Saracatinib) or LDHA (with Stiripentol) alone had limited efficacy, consistent with the notion that tumors employ parallel metabolic pathways to sustain growth. However, combining these agents with RACK1 knockdown yielded synergistic anti-tumor effects in xenograft and metastasis models. These data suggest that disrupting multiple nodes of the RACK1-centered metabolic network may be more effective than targeting single pathways, a concept relevant to ongoing efforts to overcome metabolic plasticity in cancer.

In summary, this study identifies RACK1 as a master coordinator of glycolysis and the PPP in LUAD, operating through c-Src-mediated G6PD phosphorylation and competitive stabilization of LDHA. The dual targeting of these axes, in combination with RACK1 perturbation, represents a rational therapeutic strategy for this challenging malignancy.

## Supplementary information

Supplementary Figure

Supplementary Tables S1

Supplementary Tables S2

Supplementary Tables S3

Supplementary Tables S4

Supplementary Tables S5

Supplementary Tables S6

Original Data

## Data Availability

Data from publicly archive datasets are available from TCGA database. All raw data are available in supplementary materials.
