# Supplementary material for: RACK1 governs a dual metabolic switch in lung adenocarcinoma through c-Src/G6PD and TRIM21/LDHA Axes
Source: Cell Death Dis. 2026 May 29;17(1):667. doi: 10.1038/s41419-026-08887-8 (PMC13424137; doi:10.1038/s41419-026-08887-8)
Supplement: Supplementary file 1 — Supplementary Figure [file 41419_2026_8887_MOESM1_ESM.docx]

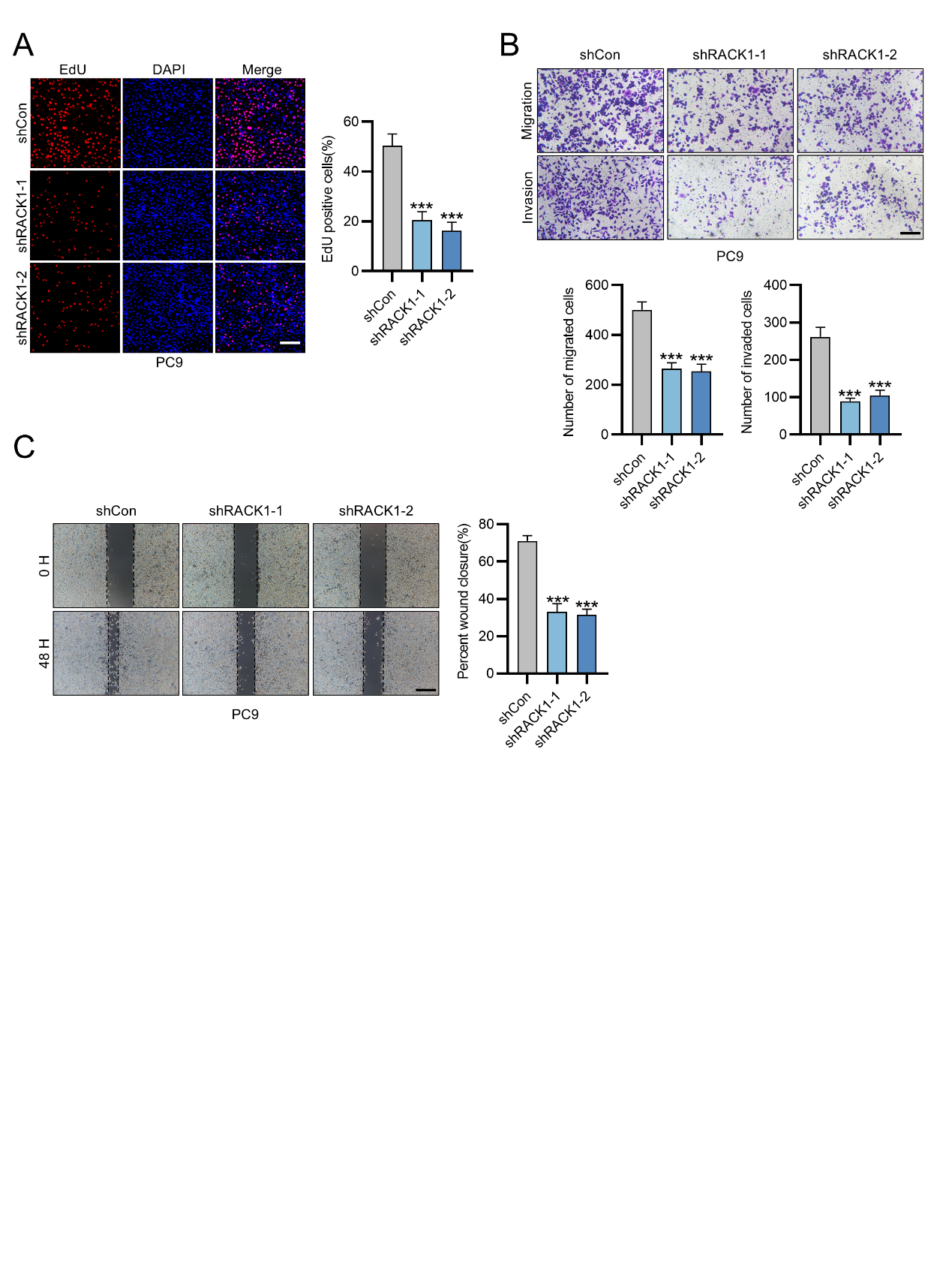
**Supplementary Figure S1. RACK1 Overexpression Enhances the Proliferation and Invasion of LUAD Cells.** (A) Representative images (left) and quantification (right) of EdU incorporation assays in PC9 cells following stable knockdown or control, indicating cell proliferation. Scale bar = 50 µm (B) Representative images of transwell migration (upper chamber) and invasion (Matrigel-coated chamber) assays in PC9 cells following stable knockdown or control. Scale bar = 100 µm. Quantitative analysis of migrated and invaded cells is shown on the bottom. (C) Representative images (left) and quantification (right) of wound-healing assays in PC9 cells following stable knockdown or control. Scale bar = 100 µm. Each experiment was performed in three independent biological replicates (n = 3). Error bars represent mean ± S.D. ***P* < 0.01, ****P* < 0.001.


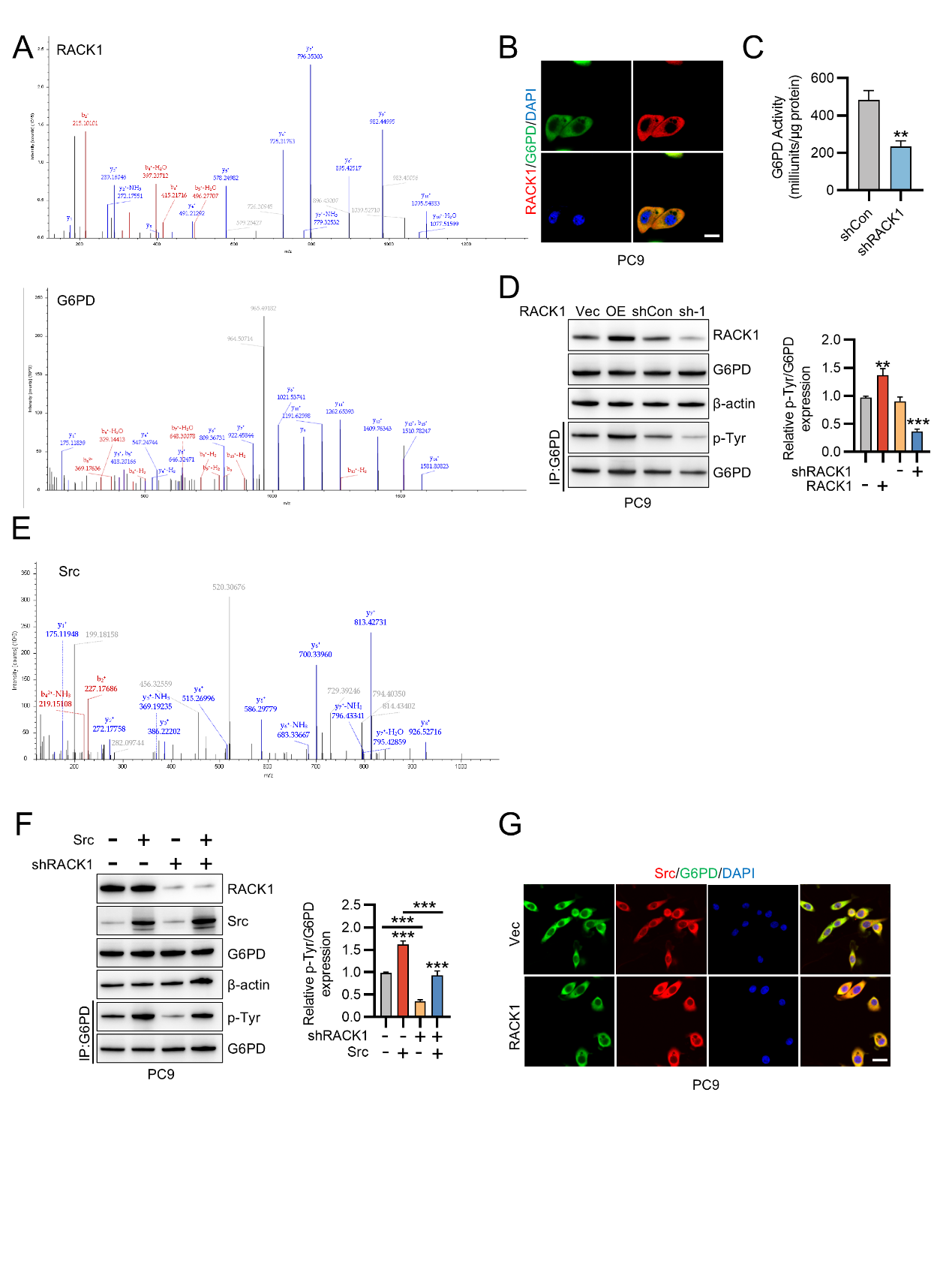
**Supplementary Figure S2. RACK1 Scaffolds c-Src to Phosphorylate and Activate G6PD.** (A) Detection of RACK1 and G6PD by liquid chromatography-mass spectrometry (LC-MS). (B) Immunofluorescence (IF) images showing co-localization of RACK1 (red) and G6PD (green) in PC9 cells. Scale bar = 10 µm. (C) G6PD enzymatic activity assay in control versus RACK1-knockdown PC9 cells. (D) Western blot analysis of G6PD tyrosine phosphorylation (p-Tyr) in PC9 cells with modulated RACK1 expression, with quantification on the right. (E) Detection of c-Src by liquid chromatography-mass spectrometry (LC-MS). (F) Western blot analysis showing that the reduction in G6PD tyrosine phosphorylation upon RACK1 knockdown is rescued by c-Src overexpression, with quantification on the right. (G) IF images showing enhanced co-localization of G6PD (green) and c-Src (red) in RACK1-overexpressing A549 cells. Scale bar = 20 µm. Each experiment was performed in three independent biological replicates (n = 3). Error bars represent mean ± S.D. ***P* < 0.01, ****P* < 0.001.


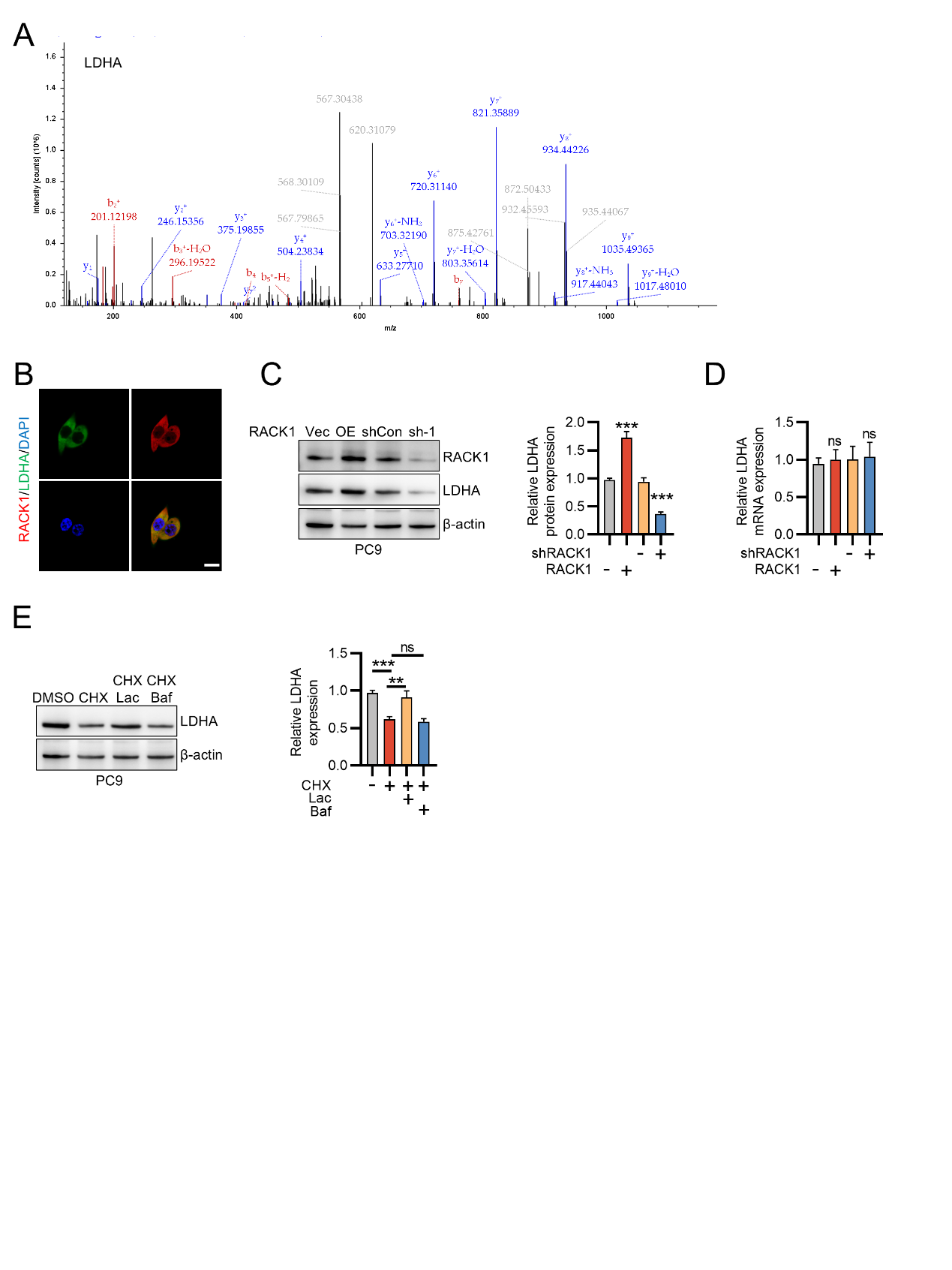


**Supplementary Figure S3. RACK1 Promotes Glycolysis by Enhancing LDHA Protein Stability.** (A) Detection of LDHA by liquid chromatography-mass spectrometry (LC-MS). (B) Immunofluorescence (IF) images showing co-localization of RACK1 (red) and LDHA (green) in PC9 cells. Scale bar = 20 µm. (C) Western blot analysis (left) and quantification (right) of LDHA protein levels in PC9 cells with RACK1 knockdown or overexpression. (D) RT-qPCR analysis of LDHA mRNA levels in PC9 cells with modulated RACK1 expression. (E) Western blot analysis of LDHA protein levels in A549 cells treated for 9 hours with the protein synthesis inhibitor cycloheximide (CHX, 4 µg/mL) in combination with the proteasome inhibitor Lactacystin (Lac, 20 µM) or the lysosome inhibitor bafilomycin A1 (Baf, 200 nM). DMSO was used as a vehicle control. Quantification is shown on the right. Each experiment was performed in three independent biological replicates (n = 3). Statistical significance was determined by a two-tailed unpaired Student's t-test. Error bars represent mean ± S.D. ***P* < 0.01, ****P* < 0.001.


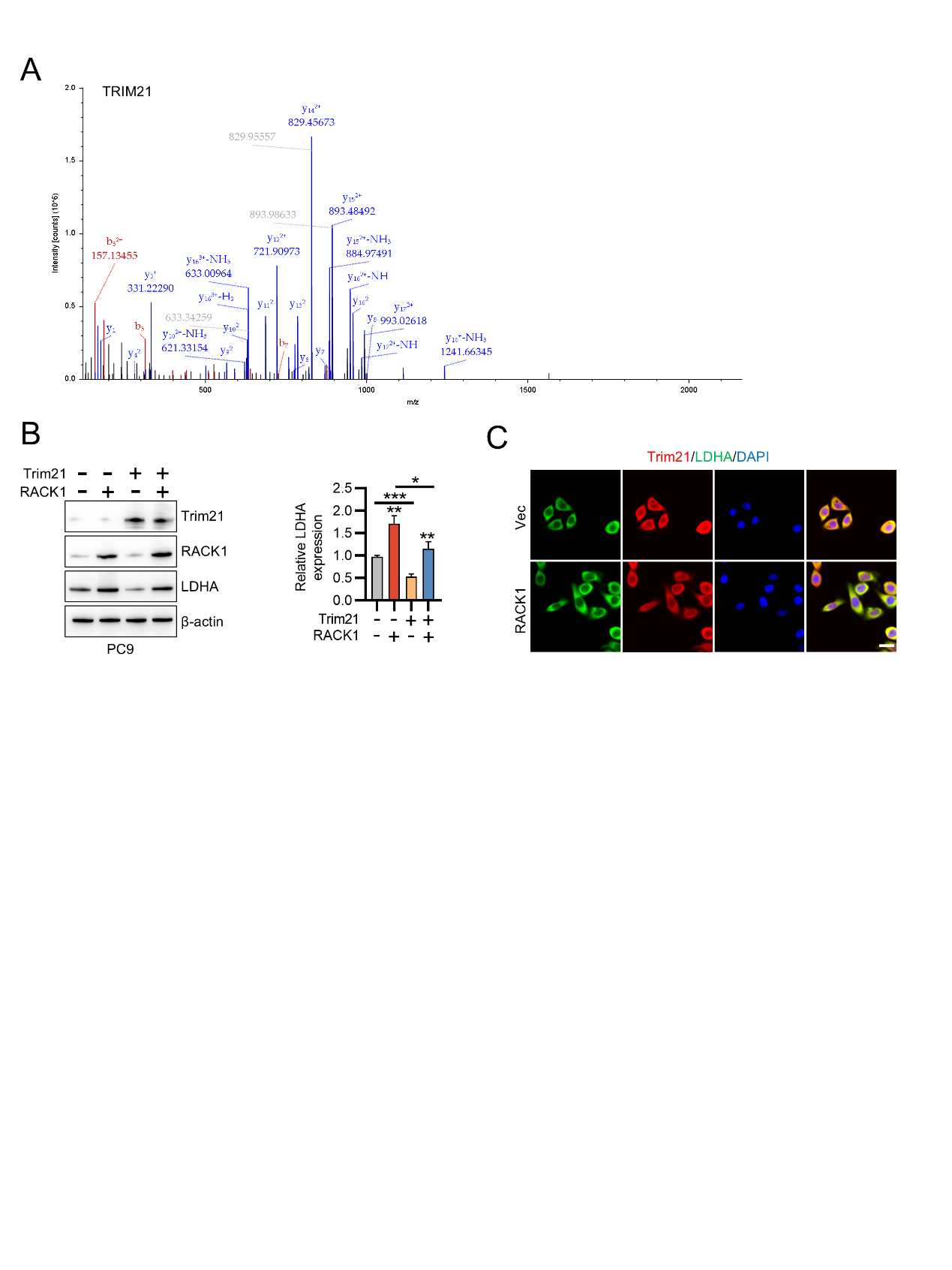
**Supplementary Figure S4. RACK1 and TRIM21 Competitively Bind to LDHA to Regulate Its Protein Stability.** (A) Detection of Trim21 by liquid chromatography-mass spectrometry (LC-MS). (B) Western blot analysis (left) and quantification (right) of LDHA protein levels in PC9 cells with TRIM21 knockdown and RACK1 overexpression. (C) Immunofluorescence (IF) images showing reduced co-localization of LDHA (green) and TRIM21 (red) in RACK1-overexpressed PC9 cells. Scale bar = 10 µm. Each experiment was performed in three independent biological replicates (n = 3). Statistical significance was determined by a two-tailed unpaired Student's t-test. Error bars represent mean ± S.D. **P* < 0.05, ***P* < 0.01, ****P* < 0.001.


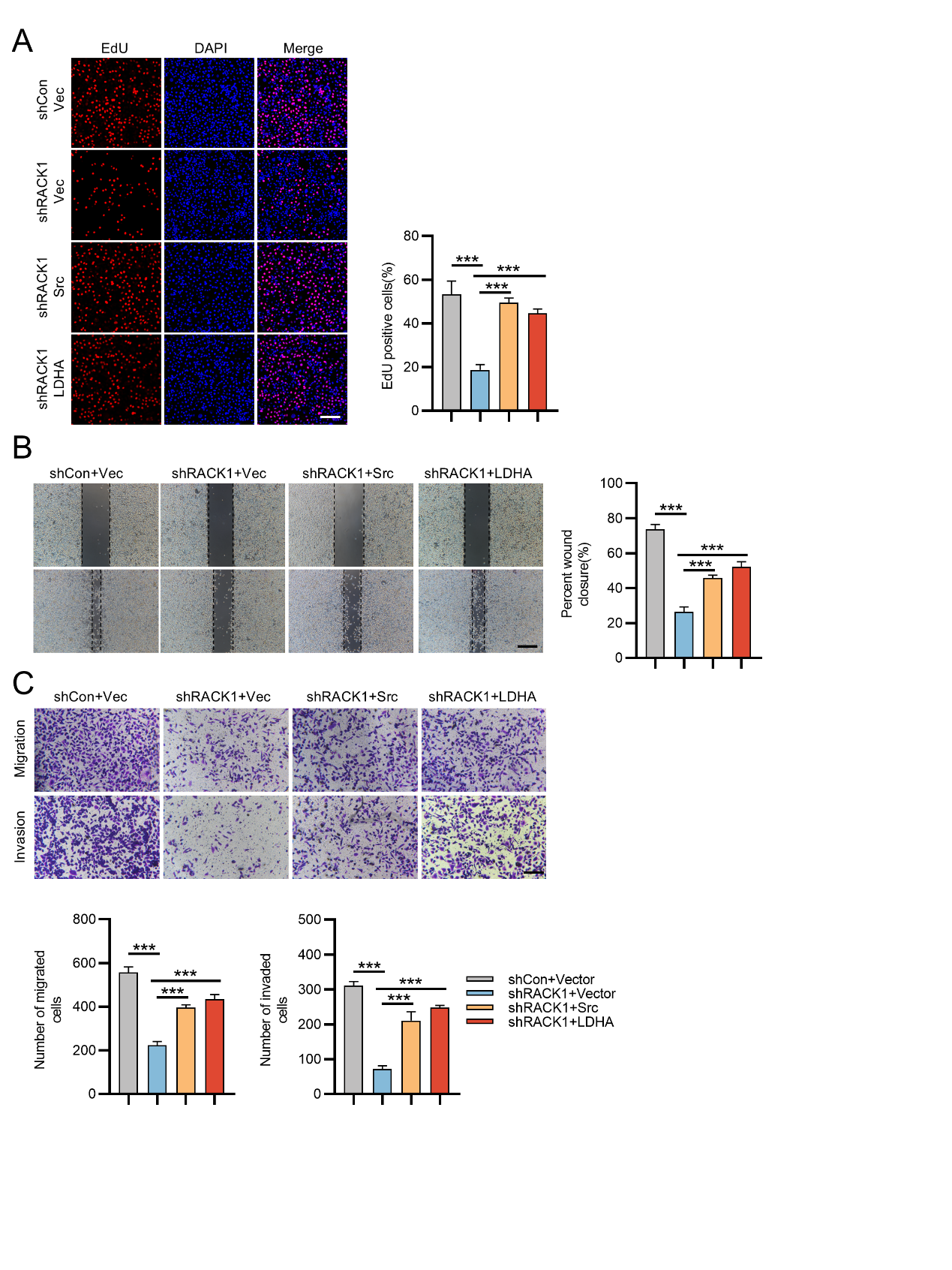
**Supplementary Figure S5. RACK1 Promotes LUAD Malignancy by Coordinately Enhancing Glycolysis via LDHA and the Pentose Phosphate Pathway via G6PD.**

(A) Representative images (left panel) and quantification (right panel) of EdU incorporation assays in control and RACK1-knockdown PC9 cells, showing that the proliferation deficit is rescued by re-expression of c-Src or LDHA. Scale bar = 50 µm. (B) Representative images (left panel) and quantification right panel) of wound-healing assays in control and RACK1-knockdown PC9 cells, showing that the migration defect is rescued by re-expression of c-Src or LDHA. Scale bar = 100 µm. (C) Representative images of transwell migration (upper chamber) and invasion (Matrigel-coated chamber) assays in control and RACK1-knockdown PC cells, showing that the invasive and migratory deficits are rescued by re-expression of c-Src or LDHA. Scale bar = 100 µm. Each experiment was performed in three independent biological replicates (n = 3). Statistical significance was determined by a two-tailed unpaired Student's t-test. Error bars represent mean ± S.D. ***P* < 0.01, ****P* < 0.001.


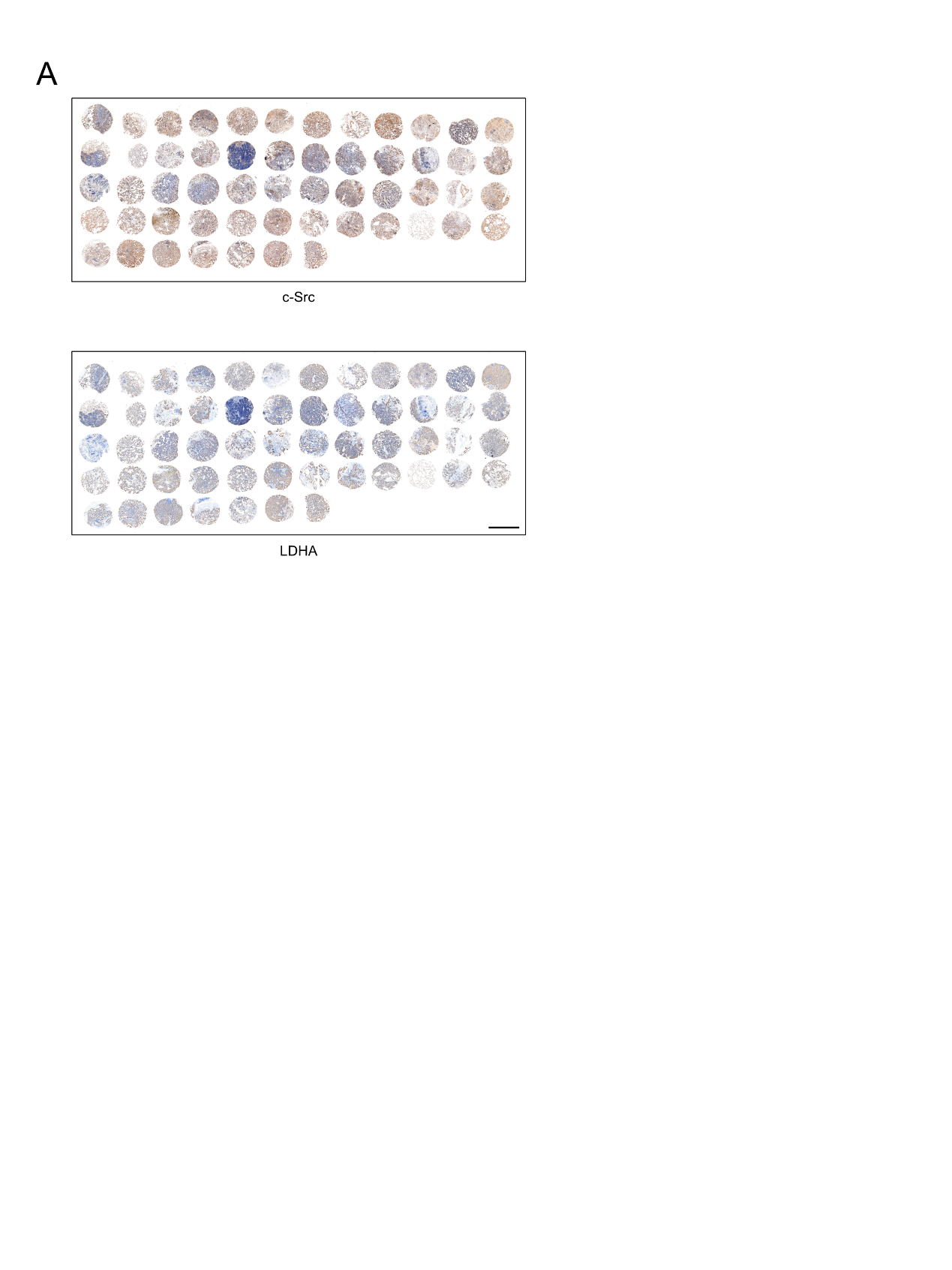
**Supplementary Figure S6. Targeting the RACK1-c-Src-G6PD and RACK1-LDHA Axes as a Therapeutic Strategy Against LUAD.** (A) Representative images of c-Src and LDHA immunohistochemical (IHC) staining on a tissue microarray (TMA) containing 55 LUAD cases. Scale bar = 2000 µm
