# Supplementary material for: RACK1 governs a dual metabolic switch in lung adenocarcinoma through c-Src/G6PD and TRIM21/LDHA Axes
Source: Cell Death Dis. 2026 May 29;17(1):667. doi: 10.1038/s41419-026-08887-8 (PMC13424137; doi:10.1038/s41419-026-08887-8)
Supplement: Supplementary file 2 — Supplementary Tables S1 [file 41419_2026_8887_MOESM2_ESM.docx]

**Supplementary Table S1**

Table 1. Clinicopathological characteristics of 21 patients with LUAD

| **Characteristics** |  | **Number** | **No. of patients** | | **P-value** |
| --- | --- | --- | --- | --- | --- |
|  |  |  | **Early**  **stage** | **Advanced**  **stage** |  |
| Age (y) | ≥60  <60 | 10  11 | 5  5 | 5  6 | >0.9999 |
| Gender | Male  Female | 9  12 | 5  5 | 4  7 | 0.6699 |

^i^Early stage includes stage I and II; Advanced stage includes stage IIIb and IIIc in pathological stage
