# Supplementary material for: RACK1 governs a dual metabolic switch in lung adenocarcinoma through c-Src/G6PD and TRIM21/LDHA Axes
Source: Cell Death Dis. 2026 May 29;17(1):667. doi: 10.1038/s41419-026-08887-8 (PMC13424137; doi:10.1038/s41419-026-08887-8)
Supplement: Supplementary file 3 — Supplementary Tables S2 [file 41419_2026_8887_MOESM3_ESM.docx]

**Supplementary Table S2**

Table 2. Clinicopathological characteristics of 55 patients with LUAD

| **Characteristics** |  | **Number** | **No. of patients** | | **P-value** |
| --- | --- | --- | --- | --- | --- |
|  |  |  | **Early**  **stage** | **Advanced**  **stage** |  |
| Age (y) | ≥60  <60 | 29  26 | 11  14 | 18  12 | 0.2850 |
| Gender | Male  Female | 25  30 | 9  16 | 16  14 | 0.2781 |

^i^Early stage includes stage I and II; Advanced stage includes stage IIIb and IIIc in pathological stage
