# Supplementary material for: RACK1 governs a dual metabolic switch in lung adenocarcinoma through c-Src/G6PD and TRIM21/LDHA Axes
Source: Cell Death Dis. 2026 May 29;17(1):667. doi: 10.1038/s41419-026-08887-8 (PMC13424137; doi:10.1038/s41419-026-08887-8)
Supplement: Supplementary file 4 — Supplementary Tables S3 [file 41419_2026_8887_MOESM4_ESM.docx]

**Supplementary Table S3**

**Detailed information forprimers and reagents**

| **Primers for PCR and real time PCR** | | | |
| --- | --- | --- | --- |
| Primers | Sequences-F (5’ → 3’) | Sequences-R (5’ → 3’) | |
| RACK1 | CGCTAGATGGTGGGGACATC | TTGTCCGTGTAGCCAGCAAA | |
| LDHA | TGCAACCAACTATCCAAGTGT | AGGGTTGCCCAAGAATAGCC | |
| β-ACTIN | CCTCGCCTTTGCCGATCC | CCATCACGCCCTGGTGC | |
|  | | | |
| **Sequences for gene knockdown** | | | |
|  | Target Sequence | | |
| shRACK1-1 | TAGCCTGTGTGGCCAATGT | | |
| shRACK1-2 | GATAACTTCTTGCTTCAGT | | |
| shRACK1-3 | TTGGGCGAGAAGCGGACAC | | |
| shLDHA | GACAACATGCACAACCTCC | | |
| shSrc | TTCACGTTGAGGCCCTTGG | | |
|  | | | |
| **Reagents** | | | |
| Regent | Source | | Cat# |
| Cycloheximide | MCE | | HY-12320 |
| MG132 | MCE | | HY-13259 |
| Lactacystin | MCE | | HY-16594 |
| Bafilomycin A1 | MCE | | HY-100558 |
| Saracatinib | MCE | | HY-10234 |
| Stiripentol | MCE | | HY-103392 |
