# Supplementary material for: RACK1 governs a dual metabolic switch in lung adenocarcinoma through c-Src/G6PD and TRIM21/LDHA Axes
Source: Cell Death Dis. 2026 May 29;17(1):667. doi: 10.1038/s41419-026-08887-8 (PMC13424137; doi:10.1038/s41419-026-08887-8)

Figure 1

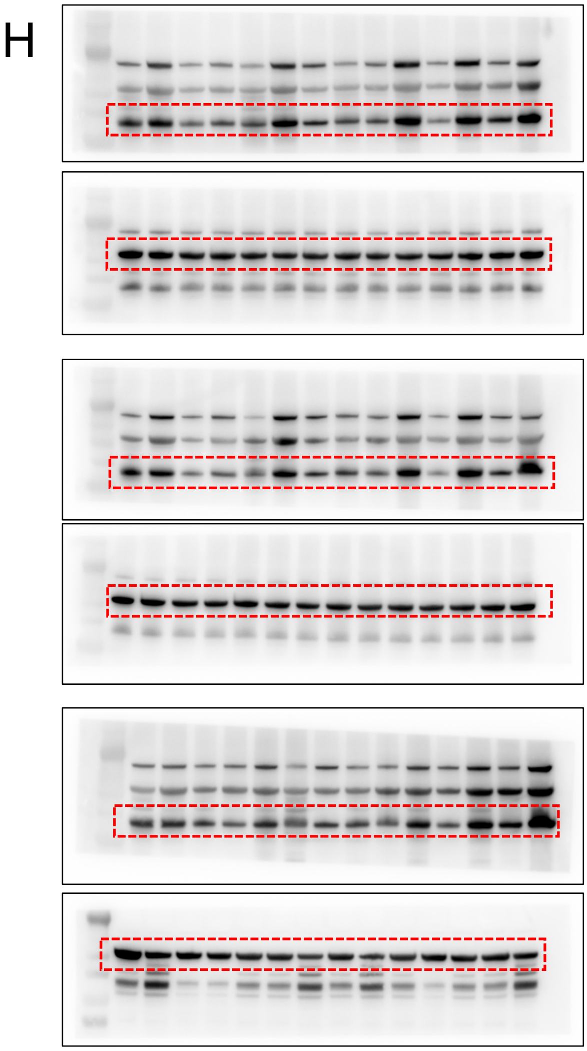

Figure 2

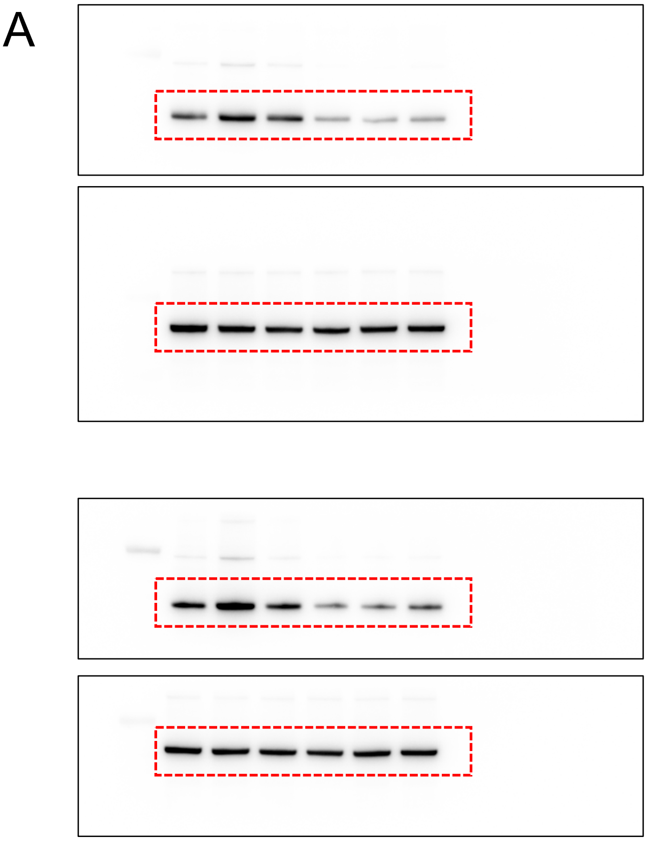

Figure 4

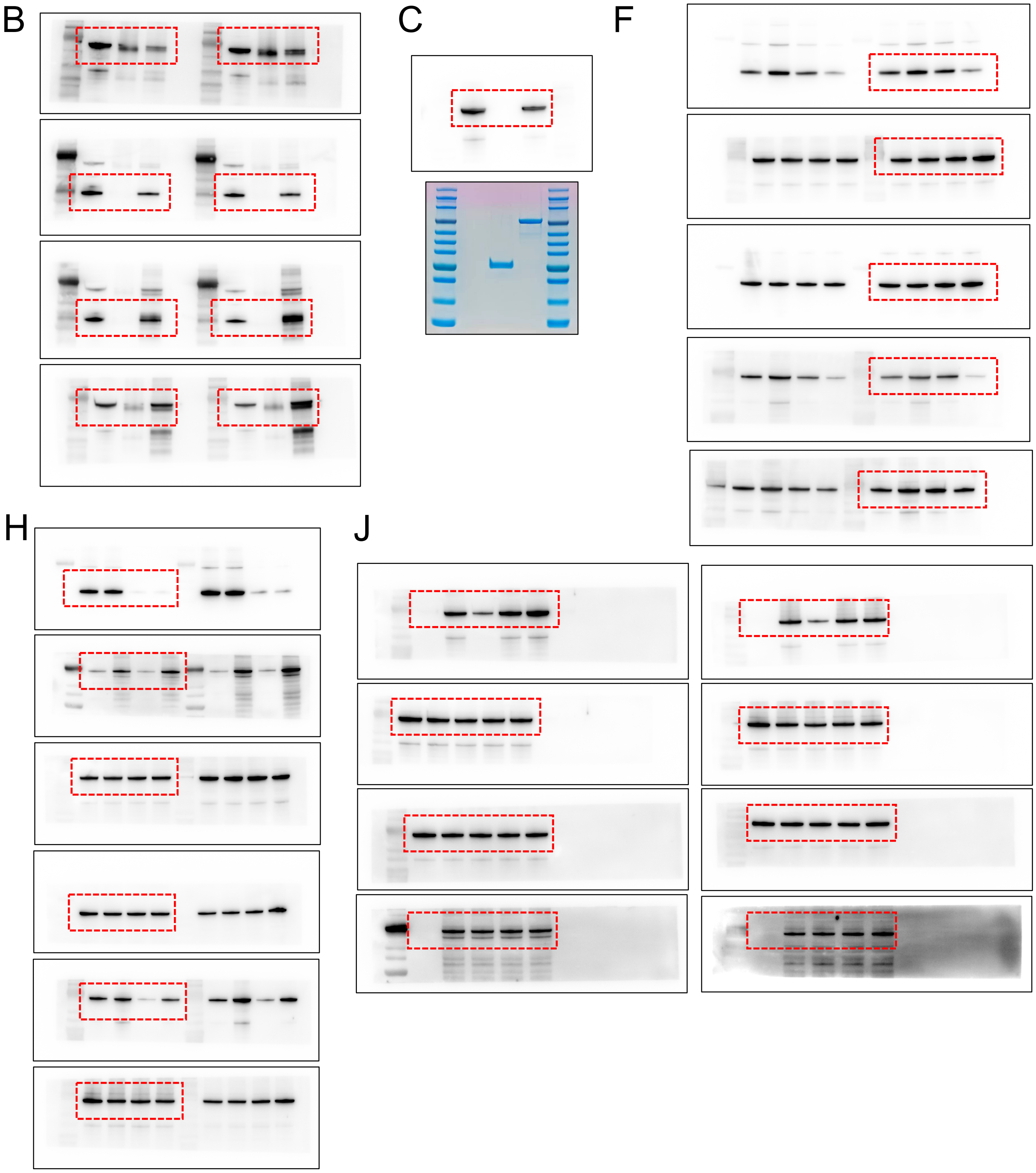

Figure 4

L

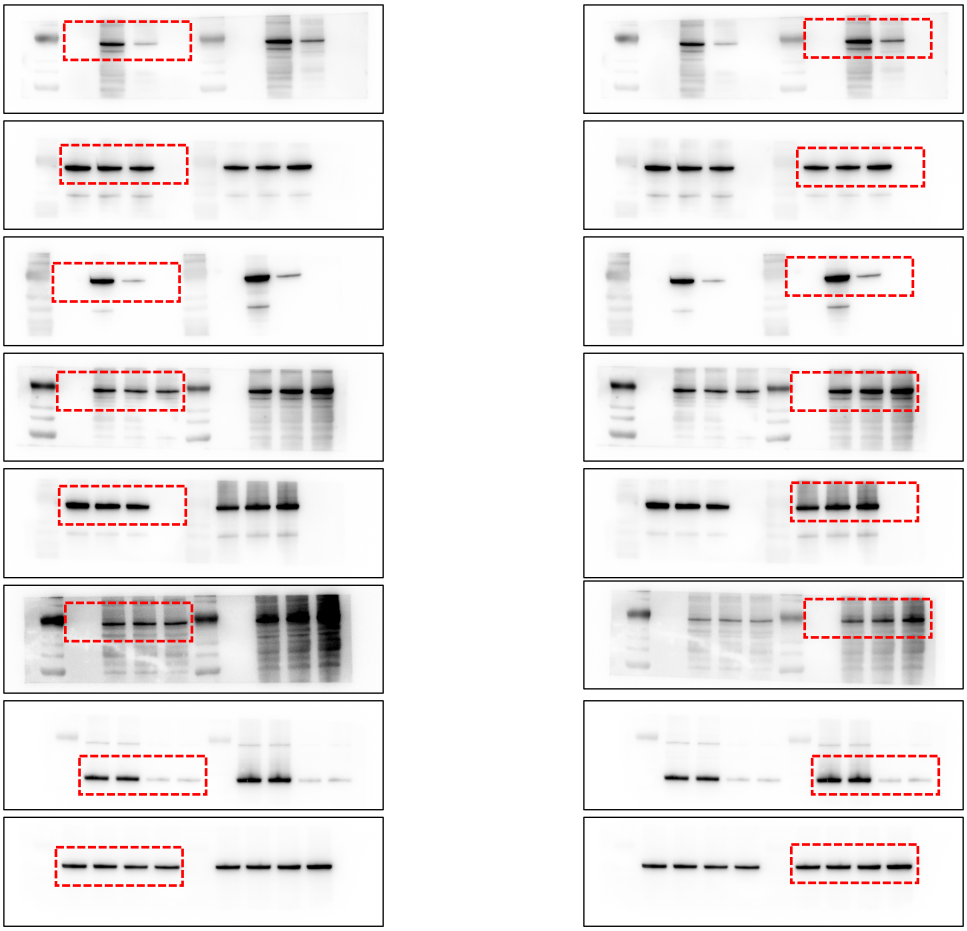

Figure 5

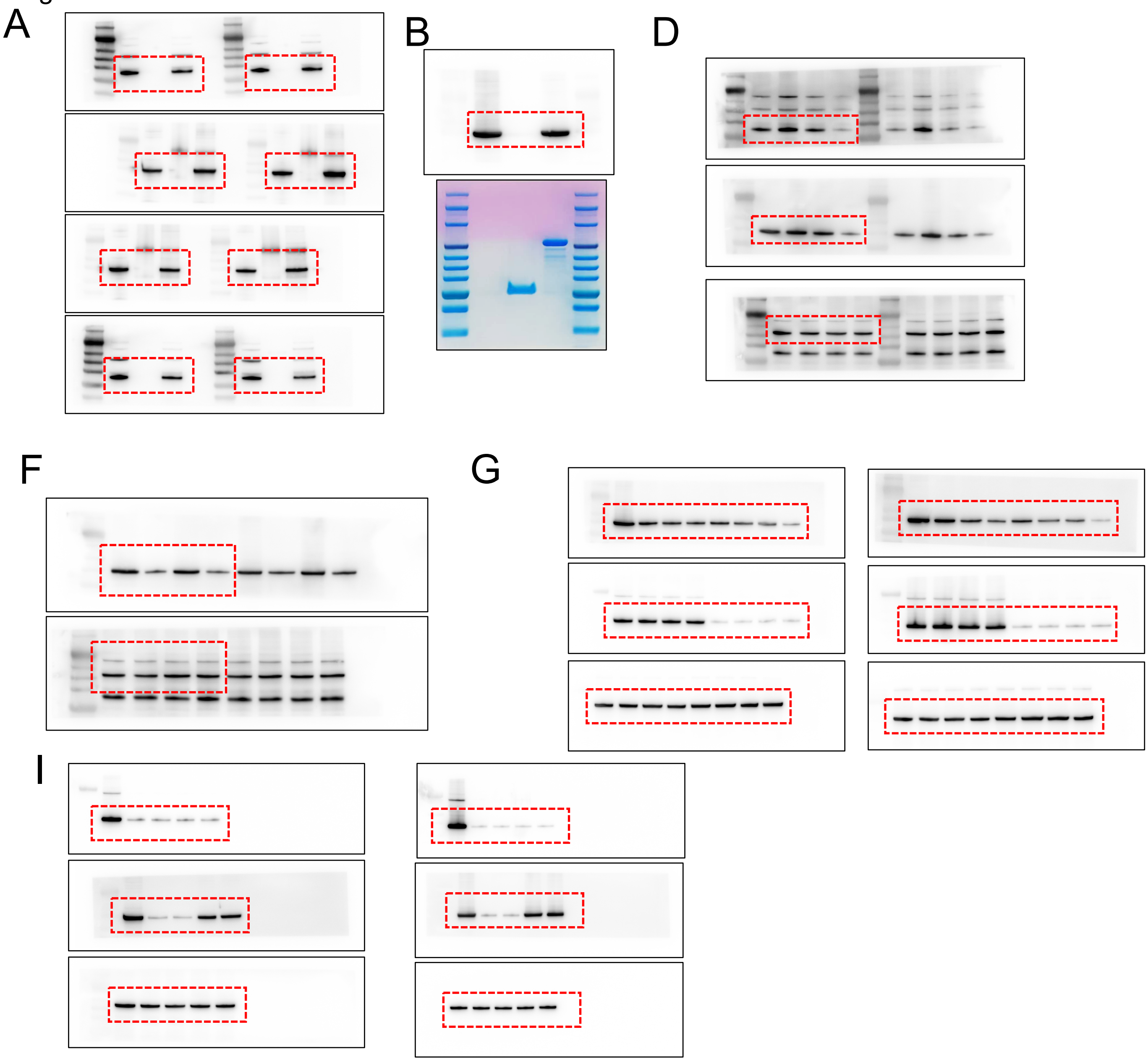

Figure 5

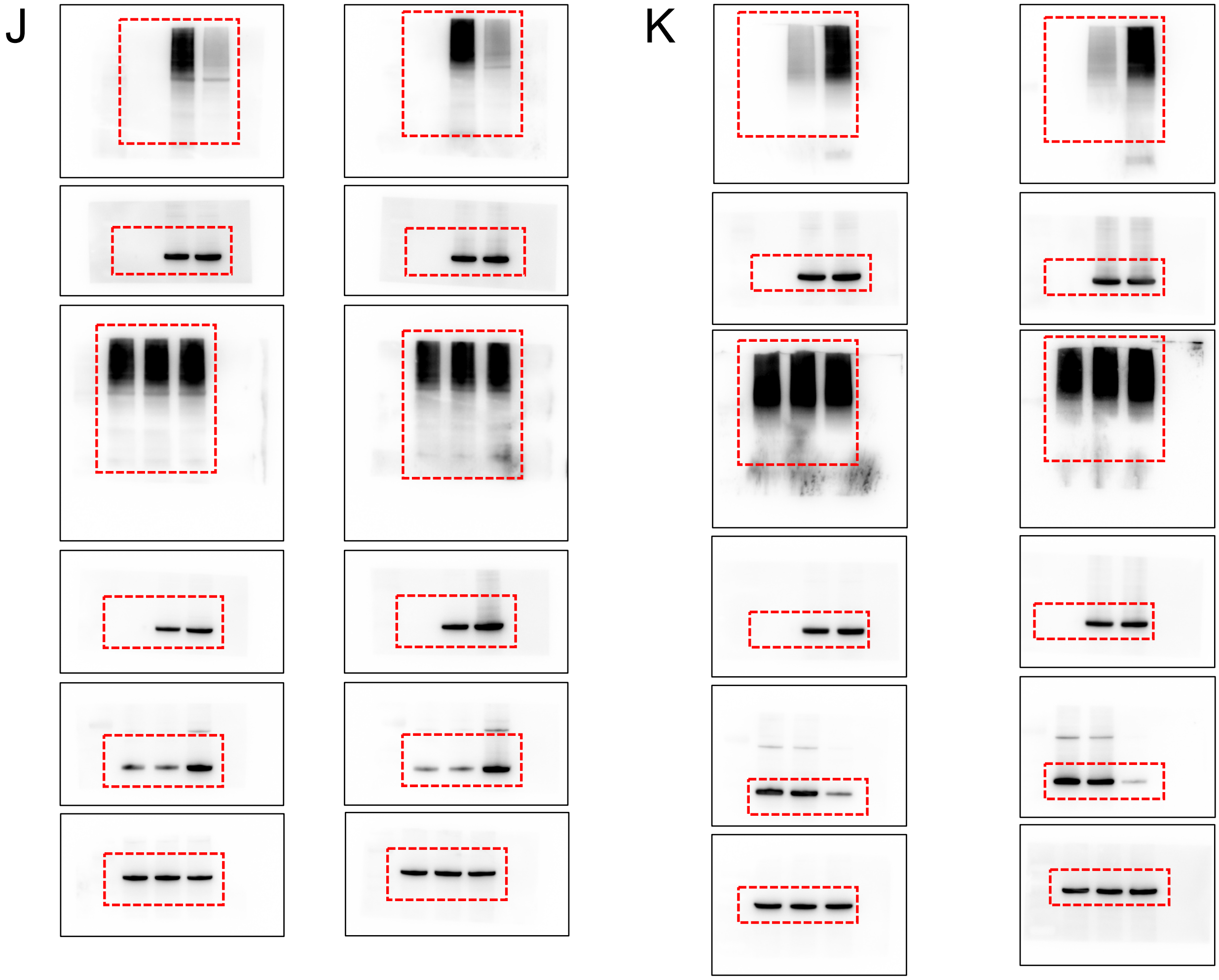

Figure 6

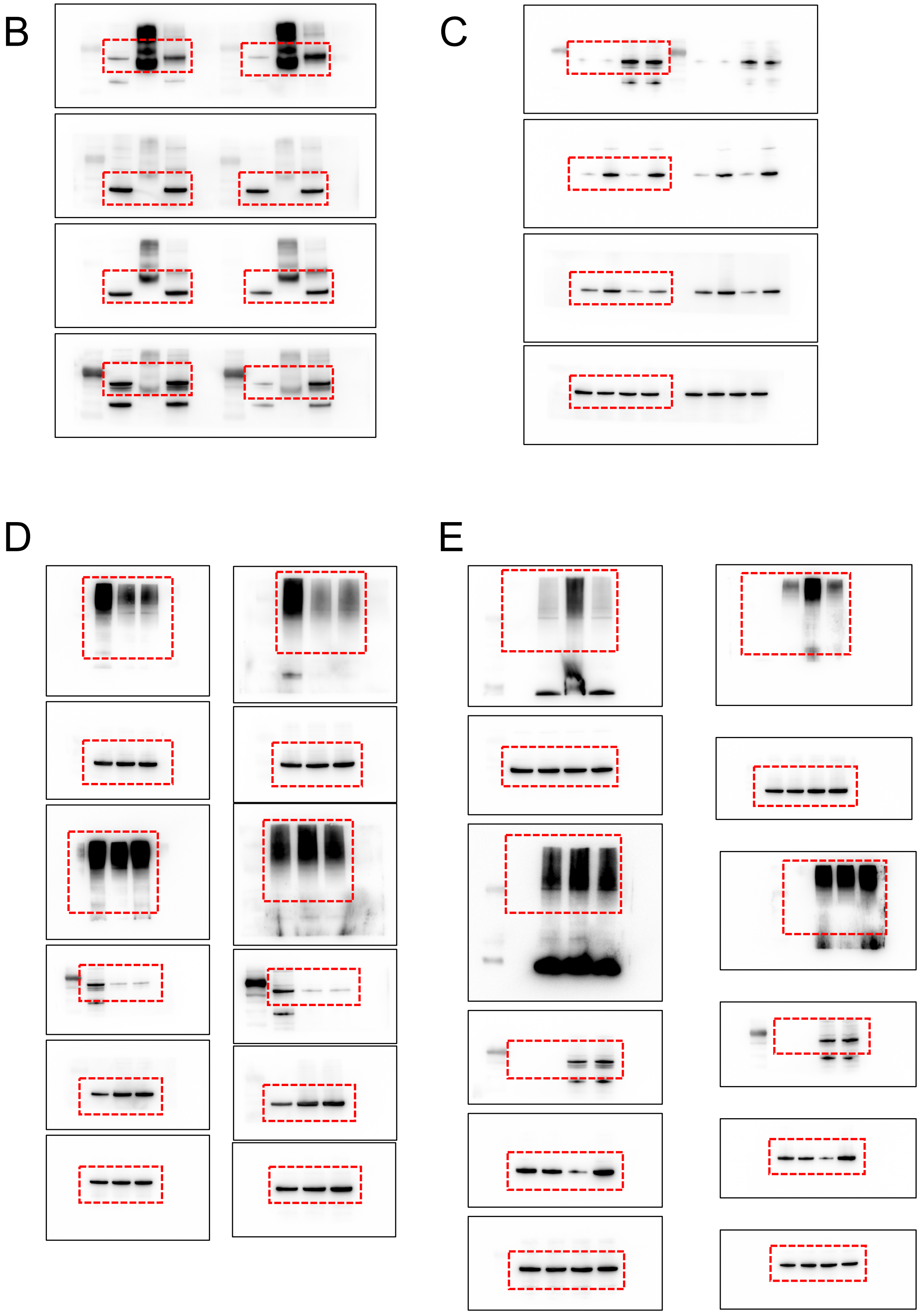

Figure 6

F

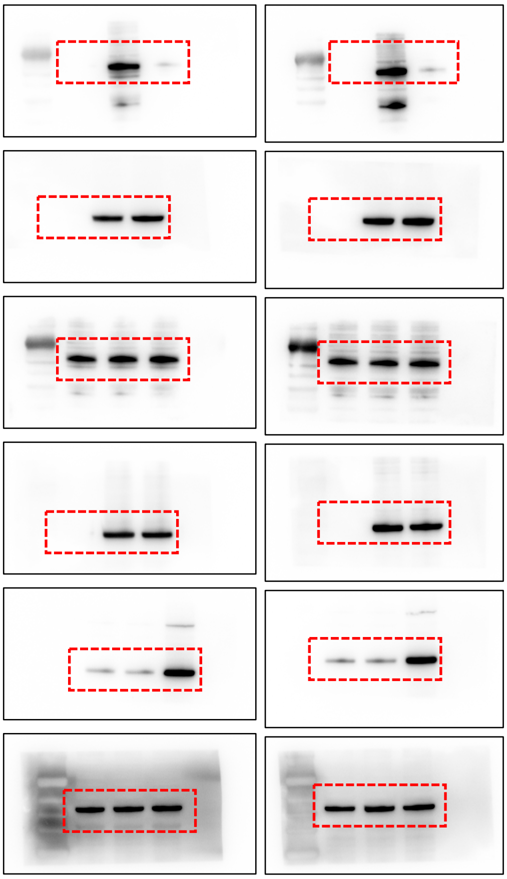

G

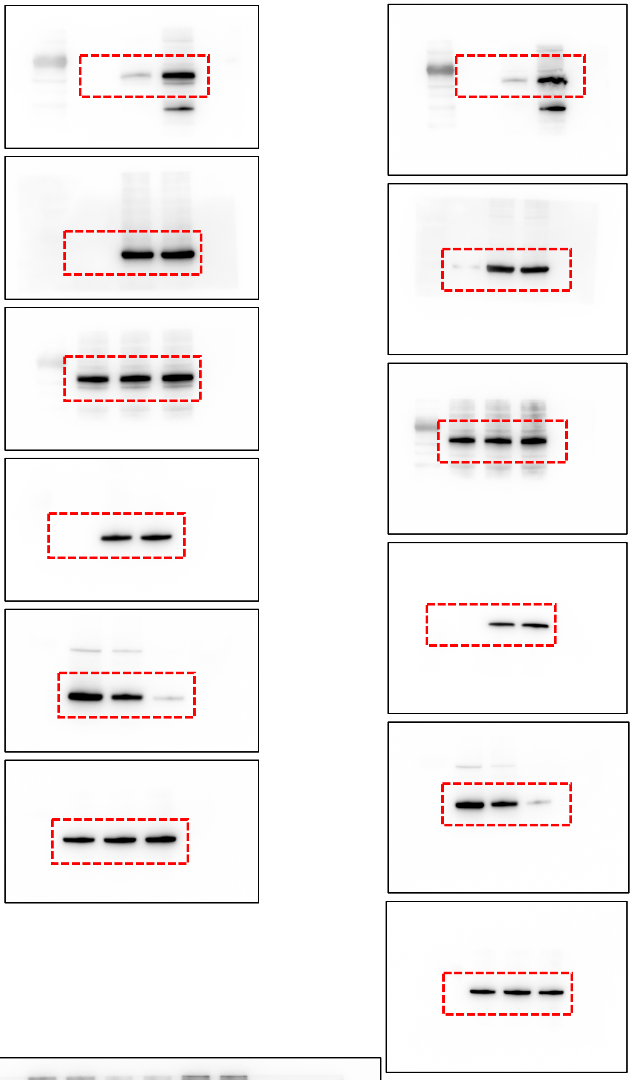

H

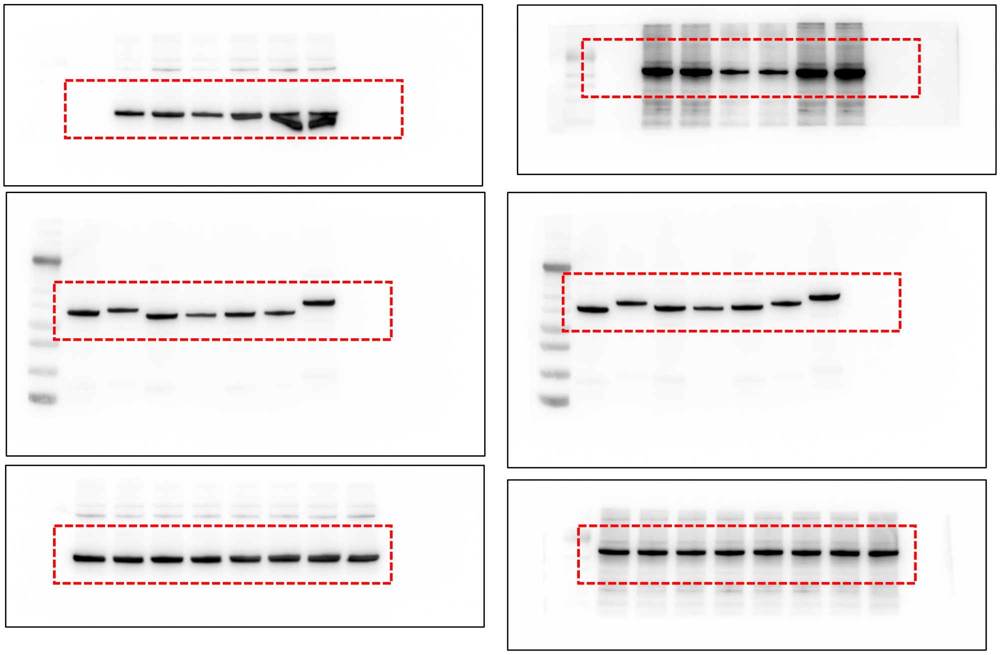

Supplementary figure 2

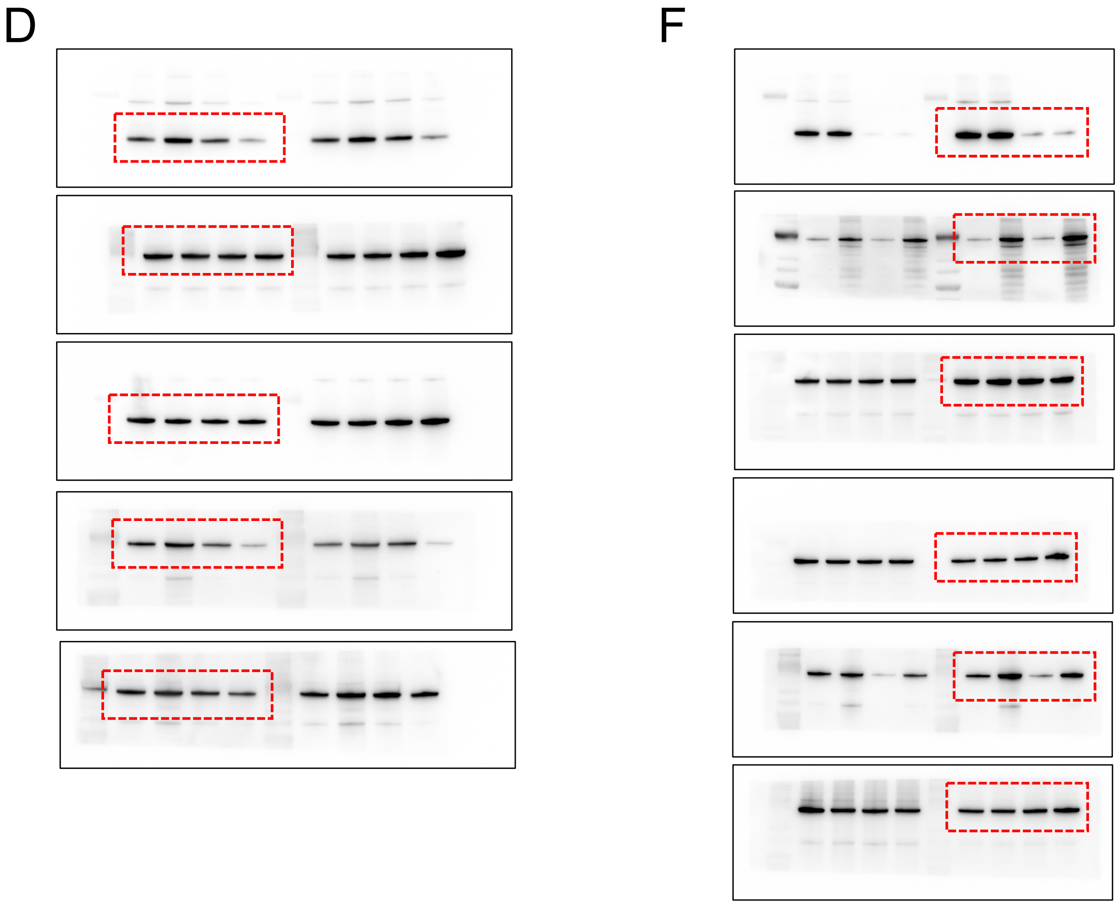

Supplementary figure 3

C

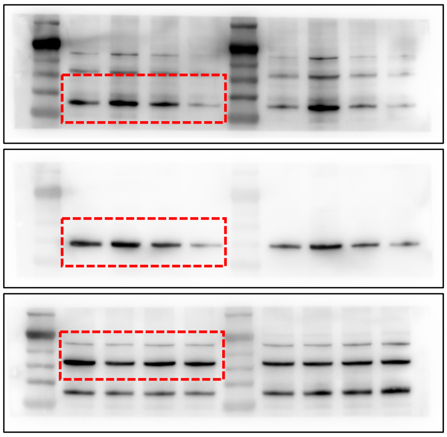

E

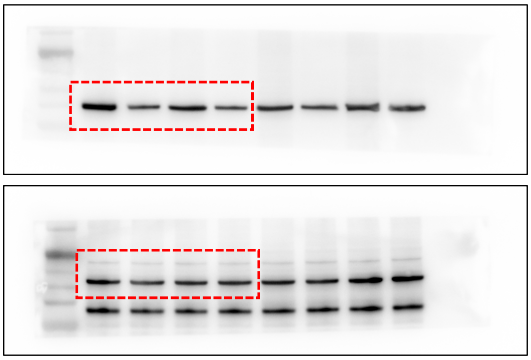

Supplementary figure 4

B

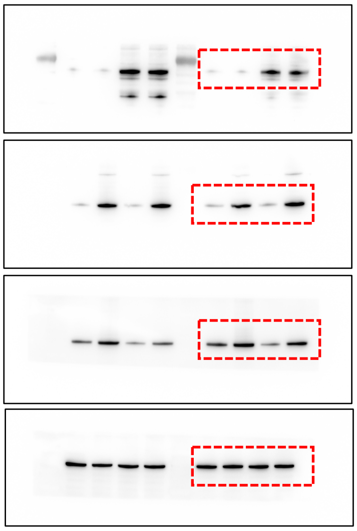

Supplement: Supplementary file 8 — Original Data [file 41419_2026_8887_MOESM8_ESM.pdf]
